# The Non‐Canonical ChREBPα Activity Suppresses the Activation of Hepatic Stellate Cells and Liver Fibrosis by Antagonizing TGF‐β‐E2F1 Axis

**DOI:** 10.1002/advs.202415032

**Published:** 2025-06-23

**Authors:** Jian Zhang, Yuee Zhao, Gauthami Pulivendala, Qing Zhang, Liangyou Rui, Jiashi Gao, Huiwen Wang, Gary Zhang, Alli Nuotio‐Antar, Xin Tong, Lei Yin

**Affiliations:** ^1^ Department of Molecular & Integrative Physiology University of Michigan Medical School Ann Arbor MI 48105 USA; ^2^ Caswell Diabetes Institute University of Michigan Medical School Ann Arbor MI 48105 USA; ^3^ Department of Infectious Diseases Xiangya Hospital Central South University Changsha Hunan Province 410008 P. R. China; ^4^ Department of Nephrology Hunan Key Laboratory of Kidney Disease and Blood Purification The Second Xiangya Hospital Central South University Changsha Hunan Province 410011 P. R. China; ^5^ Sylvester cancer center University of Miami Miami FL 33136 USA; ^6^ Department of Infectious Diseases The Second Xiangya Hospital Central South University Changsha Hunan Province 410008 P. R. China; ^7^ Department of Infection Control Center Xiangya Hospital Central South University Changsha Hunan Province 410008 P. R. China; ^8^ Children Nutrition Research Center Department of Pediatrics Baylor College of Medicine Houston TX 77030 USA

**Keywords:** ChREBPα, E2F1, hepatic stellate cells, liver fibrosis, TGF‐β signaling

## Abstract

Sustained activation of hepatic stellate cells (HSCs) drives liver fibrosis in response to chronic liver injury and inflammation. It is reported that profibrogenic signals released from stressed/injured hepatocytes evoke fibrogenic responses in HSCs. However, intrahepatocyte players that modulate such cell‐to‐cell communications remain poorly defined. In this study, hepatic ChREBPα is found to be reduced in mouse models of chemical‐induced liver fibrosis as well as in three groups of human patients with liver fibrosis. *Chrebpα‐LKO* mice are highly sensitive to both chemical (CCL4 and TAA) and bile duct ligation (BDL)‐induced liver injury and developed more advanced liver fibrosis without affecting liver lipid content. Hepatocyte ChREBPα overexpression suppressed the activation of HSCs in an in vitro medium transfer experiment in part via inhibiting the expression of profibrogenic factors THBS1 and CTGF. RNA‐Seq analysis revealed that E2F1, a novel effector of TGFβ‐mediated fibrogenic pathway, is highly induced in the liver of *Chrebpα‐LKO* mice. Hepatic knockdown of E2F1 ameliorated the increased liver fibrosis in mice with hepatic *Chrebpα* deficiency while reducing the expression of hepatic THBS1 and CTGF.

## Graphical abstract

Intrahepatic microenvironment greatly impacts duration and degree of HSCs activation and development of liver fibrosis. By inhibiting TGFβ‐E2F1‐driven production of profibrogenic factors (CTGF and THBS1), hepatocyte ChREBPα monitors hepatic microenvironment and suppresses the overactivation of HSCs. Thus, enhancing *Chrebpα* in hepatocytes could be a novel target for treating liver fibrosis during the pathogenesis of chronic liver diseases.

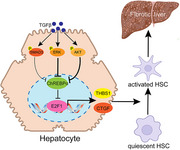

## 1 Introduction

The progression of chronic liver diseases (CLD), irrespective of etiology, involves chronic hepatocyte injury, augmented inflammation, and sustained fibrosis in the liver.^[^ ^1^ ^]^ Liver fibrosis is featured as excessive accumulation of extracellular matrix (ECM) due to its overproduction and insufficient degradation.^[^ ^2^ ^]^ Hepatic stellate cells (HSCs) constitute most of the ECM‐producing fibroblasts.^[^ ^2^, ^3^ ^]^ In healthy livers, HSCs are quiescent and function primarily as the depot of retinoids. In response to acute liver injury or inflammation, they become highly proliferative and fibrogenic with the production of collagen. The fibrotic process could be reversed once injury or inflammation is resolved. However, during chronic injury and inflammation stress, HSCs become persistently activated, leading to excessive production of ECM and eventually the scarring of liver.^[^ ^4^ ^]^ How to block or reverse HSCs activation has become the pivotal target for treating liver fibrosis.

Signals from hepatocytes, immune cells, and liver sinusoidal cells (LSECs) communicate with HSCs and modulate activation of HSCs via either paracrine mechanism or direct cell‐cell contact.^[^ ^5^ ^]^ As the dominant cell type in the liver, hepatocytes can react to fibrogenic signals from TGFβ, PDGFβ, and IL‐11 to synthesize and release various pro‐fibrogenic factors into local microenvironment.^[^ ^6^ ^]^ Abundant evidence implicates that the TGF‐β signaling drives tissue fibrosis during chronic inflammation.^[^ ^7^ ^]^ In the healthy liver, the TGF‐β signaling is transiently activated to promote tissue regeneration after acute injury and will be terminated due to the activation of multiple negative feedback loops once the repair is complete.^[^ ^7^, ^8^ ^]^ However, during CLD, persistent activation of TGF‐β signaling results in chronic activation of HSCs and fibrogenesis. Hepatocytes respond to TGF‐β via both the canonical SMAD and the non‐canonical SMAD‐independent pathways. Inhibition of hepatocyte TGF‐β signaling by deletion of either *Tgf‐β receptor 2* (*Tgf‐β2*) or *Smad4* was shown to protect mice from liver inflammation and fibrosis.^[^ ^9^ ^]^ These findings suggest that inhibition of hepatocyte TGF‐β signaling could be a viable option for reversing liver fibrosis. However, given the essential function of TGF‐β in liver regeneration, direct inhibition of TGF‐β may lead to untoward consequences, raising the need to uncover and target more specific downstream effectors of hepatocyte TGF‐β signaling to prevent or treat liver fibrosis.

The bHLH/ZIP transcription factor ChREBPα is abundantly expressed in hepatocytes and transactivates lipogenic enzymes in response to glucose/fructose intake.^[^ ^10^ ^]^ Liver ChREBPα expression was found to be induced by the intake of high‐carbohydrate diets but paradoxically markedly downregulated in both mouse and human MASH liver samples.^[^ ^11^ ^]^ Most recently, we reported that hepatocyte‐specific *Chrebpα* deletion sensitizes mice to early‐onset and augments liver injury and fibrosis, whereas hepatocyte overexpression of ChREBPα does the opposite upon diet treatment.^[^ ^11b^ ^]^ Although we revealed that the hepatoprotective action of ChREBPα mainly depends on its ability to stimulate the PPARα‐dependent fatty acid oxidation, whether hepatocyte ChREBPα harbors an anti‐fibrotic function independently of its lipogenic action remains untested. Moreover, whether hepatocyte ChREBPα directly communicates with and regulates HSC activation has not been examined yet.

Here, we reported that hepatocyte deficiency of *ChREBPα* augments hepatotoxin‐induced fibrotic response in mouse liver without impacting liver steatosis, whereas liver‐specific overexpression of ChREBPα protects mice against hepatocyte injury and liver fibrosis. Mechanistically, hepatocyte ChREBPa communicates with HSCs and suppresses their activation by down‐regulating profibrogenic factors THBS1 and CTGF. Lastly, ChREBPa inhibits the TGF‐b‐induced profibrogenic pathway by repressing E2F1. In summary, we provided the first‐line evidence supporting that hepatocyte ChREBPα protects against liver fibrosis via maintaining liver microenvironment and suppressing the activation of HSCs.

## 2 Results

### 2.1 Downregulation of Hepatic ChREBPα in Response to Fibrogenic Insults

**Figure 1:**
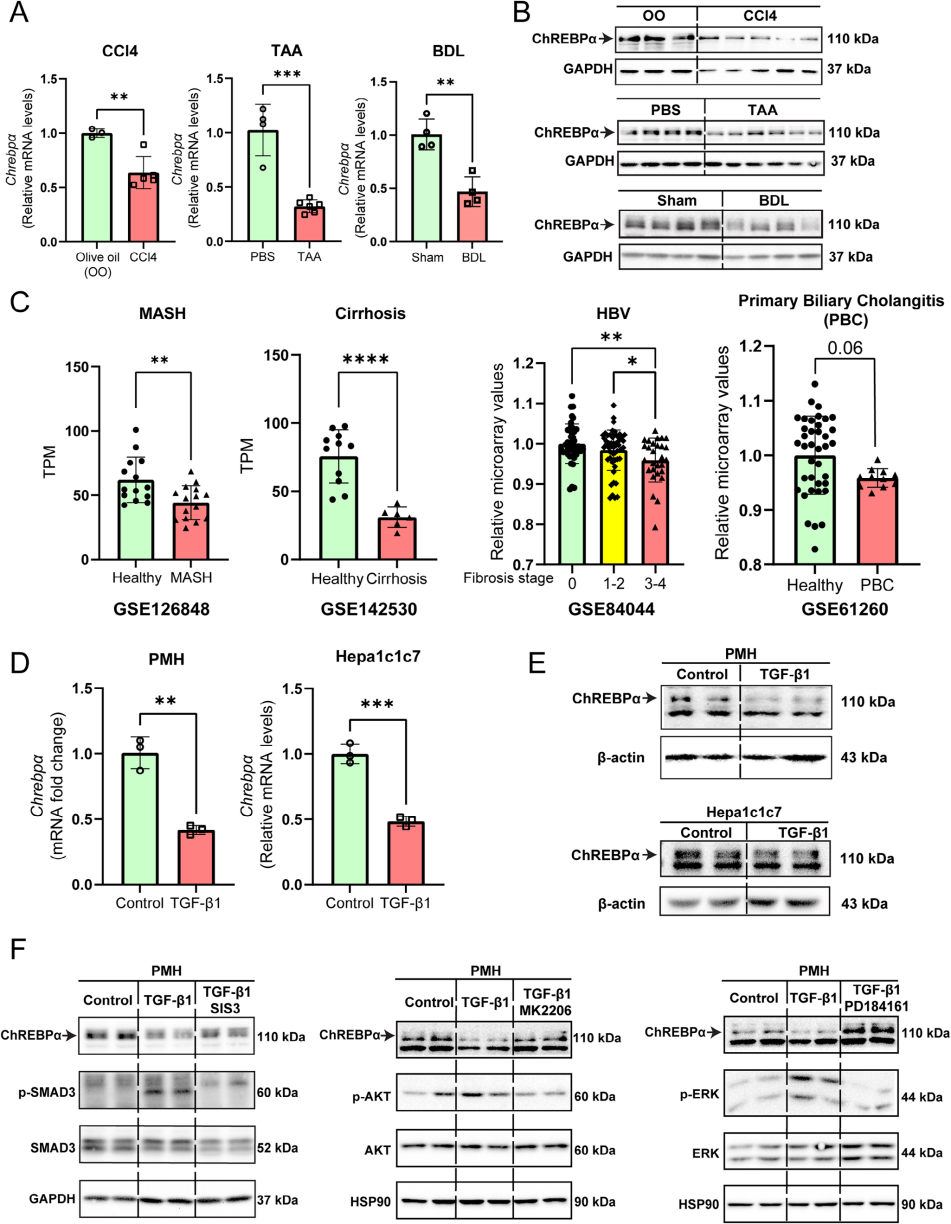
Downregulation of hepatic ChREBPα in both mouse and human liver samples with liver fibrosis. (A,B) Liver *Chrebpα* mRNA and protein expression levels in C57BL6 male mice injected with CCl4 for 4 weeks (*n* = 3, 5), TAA for 6 weeks (*n* = 4, 6), or bile duct ligation (BDL) for 7 days (*n* = 4, 4). (C) Relative expression levels of *ChREBPα* in human liver samples from RNA‐Seq data on MASH‐associated liver fibrosis (*GSE126848*) or cirrhosis (*GSE142530*) as well as from microarray data on HBV‐induced (*GSE84044*) or PBC‐induced (*GSE61260*) liver fibrosis. (D,E). *Chrebp*α mRNA and protein expression levels in PMHs and Hepa1c1c7 cells treated with TGF‐β1 (2 ng mL^−1^) (*n* = 3/group for RT‐qPCR; *n* = 2/group for western blot). (F) The protein levels of ChREBPα in PMHs treated with TGF‐β1 (2 ng mL^−1^) and inhibitor of SMAD3 (SIS3, 3 mm), inhibitor of AKT (MK‐2206, 65 nm), and inhibitor of ERK (PD18416, 100 nm). All results are shown as the mean ± SD. All the invitro experiments were repeated at least once with similar results observed. Comparisons between two groups were made using the two‐tailed Student's *t*‐test, and comparisons between three groups were made using one‐way ANOVA followed with post‐hoc Tukey test. *p < 0.05; **p < 0.01, ***p < 0.001, and ****p < 0.0001.

We previously reported a marked reduction of liver ChREBPα expression in mouse models of MASH and human liver samples with MASH,^[^ ^11b^ ^]^ raising the possibility that downregulation of liver ChREBPα is a shared feature in variety of liver fibrotic diseases, not only limited to MASH. We therefore examined liver ChREBPα in liver fibrosis induced by two different hepatotoxins: CCl4 (Carbon Tetrachloride) and TAA (Thioacetamide).^[^ ^12^ ^]^ CCl4 causes hepatocyte damage, necrosis, inflammation, and fibrosis after 4 weeks of challenge, whereas TAA has been shown to induce hepatic fibrosis via its bioactive metabolites and subsequent release of pro‐inflammatory mediators to activate HSCs.^[^ ^2^, ^13^ ^]^ Both hepatic *Chrebpα* mRNA and protein levels were reduced in CCl4 or TAA‐treated mouse livers (**Figure** **1**A,B). Bile duct ligation (BDL) is another classical experimental model for studying liver fibrosis due to excessive accumulation of bile acids, also called cholestasis.^[^ ^14^ ^]^ In this model, both hepatic *Chrebpα* mRNA and protein levels showed a similar decrease in liver tissues of mouse following BDL surgery (Figure 1A,B). Taken together, we have shown a reduced ChREBPα expression in three mouse models of liver fibrosis induced by either hepatotoxins or cholestasis.

Next, we analyzed the published GSE databases from four human chronic fibrotic liver diseases, including MASH (GSE126848),^[^ ^15^ ^]^ liver cirrhosis (GSE142530),^[^ ^16^ ^]^ HBV (GSE 84044),^[^ ^17^ ^]^ Primary Sclerosis Cholangitis (PBC) (GSE 61260).^[^ ^18^ ^]^ Consistent with our previous findings, liver *ChREBP* mRNA abundance was significantly reduced in MASH patients. Intriguingly, liver *ChREBP* mRNA was also significantly reduced in liver cirrhosis, HBV, and PBC patient samples and was inversely related to the fibrosis score in the case of HBV (Figure 1C). Taken together, these findings demonstrate that liver fibrosis is strongly associated with suppressed hepatic ChREBPα expression in human patients irrespective etiology.

So far, the upstream signaling pathways that trigger the downregulation of ChREBPα during liver fibrosis is unknown. We sought to examine whether the pro‐fibrotic cytokine TGF‐β could suppress liver ChREBPα expression due to its pivotal role in promoting liver fibrosis in response to liver injury and inflammation.^[^ ^2^, ^7^, ^19^ ^]^ Even though hepatocytes, Kupffer cells, and stellate cells are all TGFβ‐responsive, we chose to examine the ChREBPα expression in TGF‐β‐treated hepatocytes given that ChREBPα is predominantly expressed in hepatocytes and regulates lipid metabolism.^[^ ^10a^ ^]^ As shown in Figure 1D,E, TGFβ treatment significantly reduced the mRNA and protein levels of ChREBPα in both primary mouse hepatocytes (PMHs) and Hepa1c1c7 (mouse hepatoma cell line). It has been established that TGF‐β signaling regulates gene expression through both the canonical SMAD‐dependent and noncanonical SMAD‐independent pathways (via AKT, ERK, and JNK/p38).^[^ ^7^, ^20^ ^]^ We found that inhibition of TGF‐β signaling using SMAD‐specific inhibitor SIS3^[^ ^21^ ^]^ did not fully restore ChREBPα protein expression. Rather, inhibition of TGF‐b signaling using either AKTi (MK2206)^[^ ^22^ ^]^ or ERKi (PD184161)^[^ ^23^ ^]^ completely restored ChREBPα protein in the presence of TGF‐β (Figure 1F). Taken together, the suppression of ChREBPα by TGF‐β mainly requires activation of AKT and ERK pathway instead of SMAD.

### 2.2 Hepatocyte Deficiency of ChREBPα Augments Liver Injury and Fibrosis in Response to Hepatotoxin and Bile‐Duct Ligation

**Figure 2:**
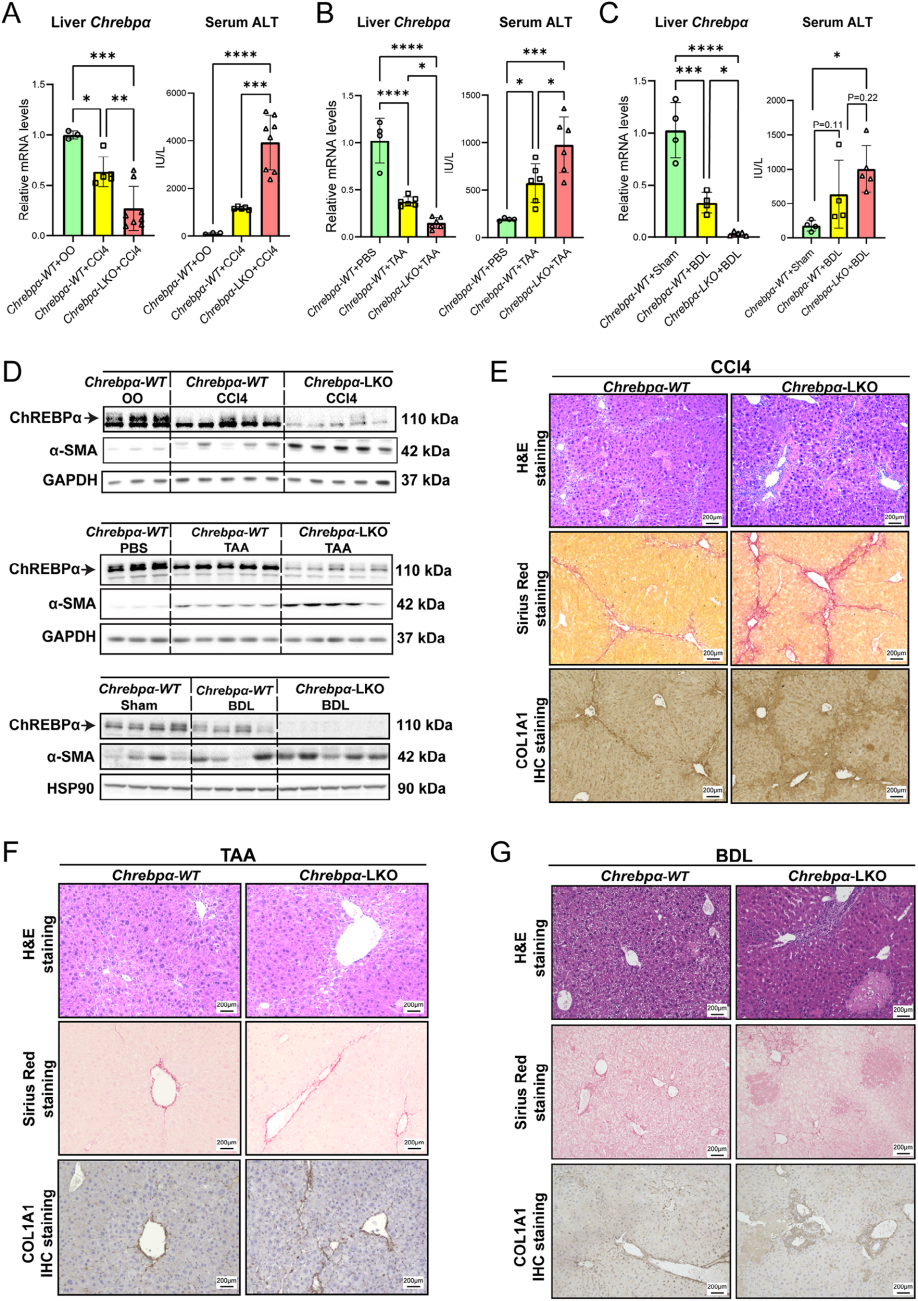
Hepatocyte‐specific deletion of *Chrebpα (Chrebpα‐LKO)* exacerbates hepatotoxin or BDL‐induced liver fibrosis in mice. *Chrebpα‐LKO* mice were generated by tail‐vein administration of AAV8‐TBG‐Cre into 8 weeks old *ChREBPα ^f/f^* male mice. The control group was injected with AAV8‐TBG‐GFP (*Chrebpα‐WT*). (A) Liver *Chrebpα* mRNA levels, serum ALT levels, liver triglycerides, and liver cholesterol levels in *Chrebpα‐WT* and *Chrebpα‐LKO* mice injected with CCl4 for 4 weeks (*n* = 3, 5, 8). (B) Liver *Chrebpα* mRNA levels and serum ALT levels in *Chrebpα‐WT* and *Chrebpα‐LKO* mice injected with TAA for 6 weeks (*n* = 4, 6, 6). (C) Liver *Chrebpα* mRNA levels and serum ALT levels in *Chrebpα‐WT* and *Chrebpα‐LKO* mice undergo BDL surgery for 7 days (*n* = 4, 4, 4). (D) Liver ChREBPα and α‐SMA protein levels in *Chrebpα‐WT* and *Chrebpα‐LKO* mice injected with CCl4 or TAA (*n* = 3, 5, 5). (E,G) *Chrebpα‐WT* and *Chrebpα‐LKO* mice liver H&E staining, Sirius Red staining, and COL1A1 IHC staining results after treatment of CCl4, TAA, or BDL. All results are shown as the mean ± SD. Differences between groups were analyzed with one‐way ANOVA and followed with post‐hoc Tukey test. *p < 0.05; **p < 0.01, ***p < 0.001, and ****p < 0.0001.

To examine the potential role of hepatocyte ChREBPα in the development of liver fibrosis, we challenged both WT and *Chrebpα‐LKO* mice with two hepatotoxin: CCl4 and TAA. We observed that both CCl4 and TAA treatments markedly increased serum ALT in WT mice. However, there was a further elevation of ALT in *Chrebp*α*‐LKO mice* (**Figure** **2**A,B), suggesting that mice with deficiency of *Chrebpα* in hepatocytes develop more severe liver injury upon chemical toxins. Of note, the liver lipid content was comparable among WT versus *Chrebpa‐LKO* following CCl4 injection, indicating that hepatocyte ChREBPα impacts hepatocyte injury independently of liver steatosis.

Next, we analyzed the severity of liver fibrosis in WT versus *Chrebpα‐LKO* mice following either CCl4 or TAA administration. One of the hallmarks of liver fibrosis, α‐SMA,^[^ ^4a^ ^]^ was further elevated in CCl4 or TAA‐injected *Chrebpα‐LKO* mice (Figure 2D). Consistent with this finding, the liver of CCl4‐treated *Chrebpα‐LKO* mice showed extensive necrotic regions by H&E staining along with extensive fibrotic scarring by Sirius Red staining and collagen deposition by COL1A1 immunohistochemistry (Figure 2E; Figure S1A, Supporting Information).^[^ ^12b^ ^]^ We observed similar responses in the liver of TAA‐treated *Chrebpα‐LKO* mice but to a lesser degree (Figure 2F; Figure S1B, Supporting Information). Of note, in both models, the mRNA levels of lipid metabolic pathways including DNL and FAO are comparable in WT versus *Chrebpα‐LKO* mice following injection of hepatotoxin (Figure S2, Supporting Information). Taken together, these findings for the first time suggest that hepatocyte ChREBPα protects against hepatotoxin‐induced liver injury and fibrosis independently of liver lipid metabolism.

To further investigate the protective role of hepatocyte ChREBPα in liver injury and fibrosis, we challenged both WT versus *Chrebpα‐LKO* with bile duct ligation. Results showed that BDL increased serum ALT in WT mice and further elevated in *ChREBPα ‐LKO* mice (Figure 2C.) H&E staining of the liver indicated severe and diffuse necrosis in *ChREBPα‐LKO* mice, which also developed more advanced liver fibrosis, indicated by the elevated a‐SMA protein and Sirius staining and COL1A1 IHC (Figure 2D,G; Figure S1C, Supporting Information). These results support that hepatocyte *Chrebpα* deficiency aggravates BDL‐induced liver fibrosis.

### 2.3 Hepatic Chrebpα Over‐Expression Protects Against CCl4‐Induced Liver Injury and Fibrosis

**Figure 3:**
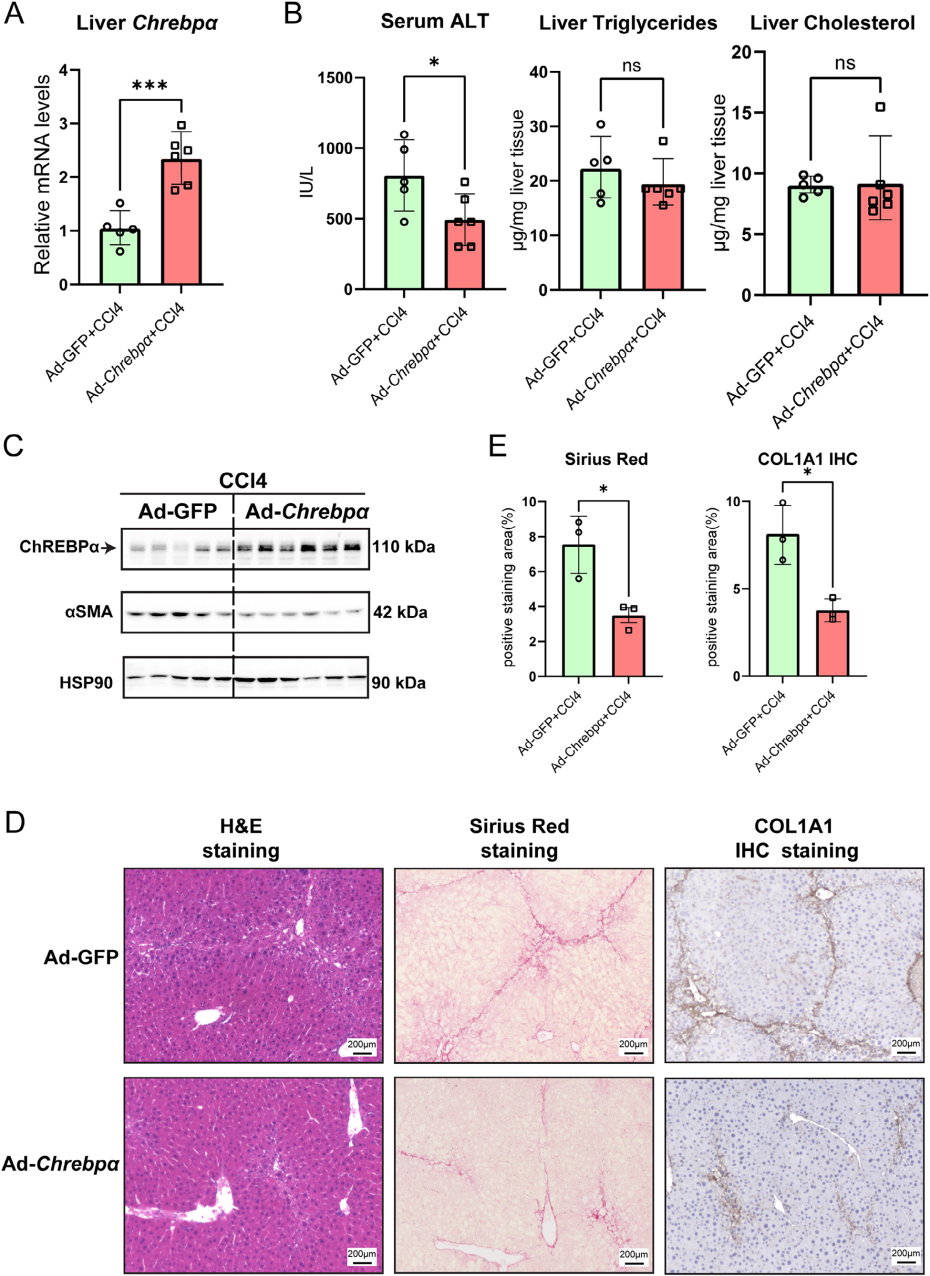
Overexpression of hepatic *Chrebpα* ameliorates CCl4‐induced liver fibrosis in mice. (A,B) Liver *Chrebpα* mRNA levels, serum ALT levels, liver triglycerides, and liver cholesterol levels in Ad‐GFP group versus Ad‐Chrebpα group after CCl4 injection for 4 weeks (*n* = 5, 6). (C) Liver ChREBPα and α‐SMA protein levels in both groups (*n* = 5, 6). (D,E) Liver H&E staining, Sirius Red staining, and COL1A1 IHC staining results and statistical results (*n* = 3/group) between these two groups. All results are shown as mean ± SD. Differences between groups were analyzed with the two‐tailed Student's *t*‐test. *p < 0.05, and ***p < 0.001.

Given our observation that hepatic *Chrebpα* is markedly reduced in fibrotic livers, we sought to examine whether transient hepatic overexpression of *Chrebpα* could protect against hepatotoxin‐induced liver fibrosis. To test this idea, we created mouse models with pre‐existing liver fibrosis by injecting WT mice with four doses of CCl4. Then, we injected Ad‐Chrebpα versus Ad‐GFP control into WT mice via tail vein to overexpress ChREBPα in the liver and continued with four additional CCl4 injections. Overexpression of hepatic ChREBPα was confirmed by immunoblotting, while α‐SMA was reduced in the liver of the same mice (**Figure** **3**A,C) along with lowered serum ALT (Figure 3B) as well as less fibrotic lesions by Sirius Red staining and reduced accumulation of COL1A1 by IHC (Figure 3D,E). In the meantime, liver total TG and cholesterol were comparable between the two groups (Figure 3B). In summary, these data demonstrate the anti‐fibrotic action of overexpressing liver ChREBPα in response to CCl4 injection without affecting liver lipid content.

### 2.4 Hepatocyte ChREBPa Suppresses Activation of HSCs In Vitro and In Vivo

**Figure 4:**
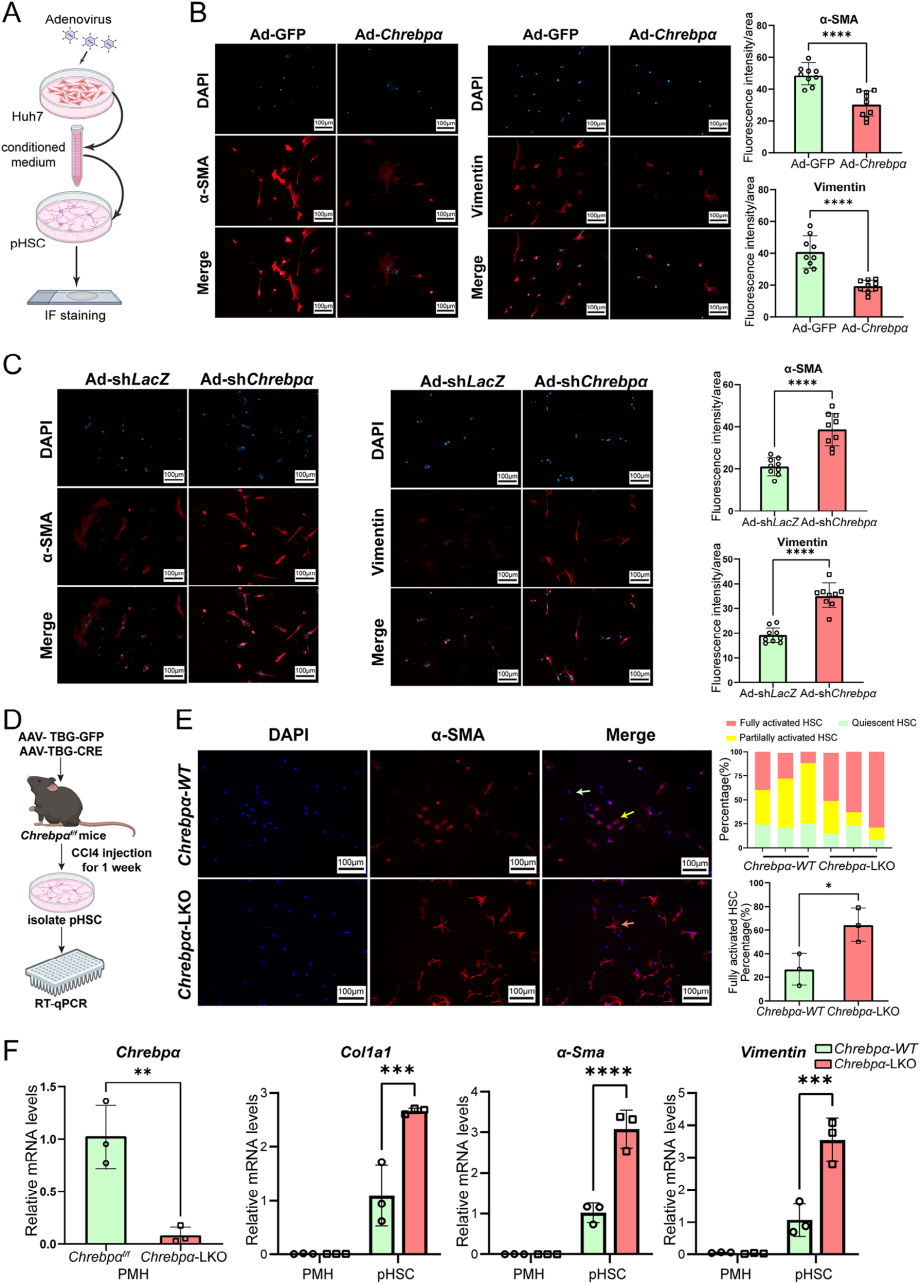
Hepatic ChREBPα suppresses HSC activation. (A) Schematic diagram of examining the crosstalk between hepatocyte and primary mouse hepatic stellate cells (pHSC) in vitro. The conditioned medium collected from adenovirus‐transduced Huh7 cells was used to incubate pmHSCs for 48 h prior to immunofluorescence (IF) staining. (B) IF staining of α‐SMA and VIMENTIN in pmHSCs treated with conditioned medium derived from either Huh7‐Ad‐GFP or Huh7‐Ad‐Chrebpα and statistical results of immunofluorescent intensity (*n* = 9 fields/group). (C) IF staining of α‐SMA and Vimentin in pHSC incubated with conditioned medium derived from either Huh7‐Ad‐shLacZ or Huh7‐Ad‐ShChrebpα (*n* = 9 fields/group). (D) Schematic outline of examining the in vivo impact of hepatocyte *Chrebp*α deficiency on activation of pmHSCs. Male Chrebpαf/f mice were injected with AAV‐TBG‐GFP (*ChREBPα ‐WT*) versus AAV‐TBG‐CRE(*ChREBPα ‐LKO*) and subsequently CCl4 injection for 1 week prior to pmHSCs isolation. (E) IF staining of α‐SMA and percentages of HSC at different activation stages were quantified (*n* = 3/group). Red arrow, fully activated HSC; yellow arrow, partially activated HSC; green arrow, quiescent HSC. (F) The mRNA level of *Chrebpα* in PMHs, and the mRNA levels of *Col1a1*, *α‐Sma* and *Vimentin* in PMHs and pmHSC from both groups. All results are shown as Mean ± SD. All the invitro experiments were repeated at least once with similar results observed. Differences between groups were analyzed with the two‐tailed Student's *t*‐test. *p < 0.05; **p < 0.01, ***p < 0.001, and ****p < 0.0001.

To uncover the mechanisms of how exactly hepatocyte *Chrebpα* deficiency augmented liver fibrosis, we reasoned that hepatocyte ChREBPa might communicate with HSCs to impact the duration and degree of activation of HSCs, which are the central driver of liver fibrosis.^[^ ^2^, ^4^ ^]^ To test this hypothesis, we designed a medium transfer experiment to examine the impact of manipulating hepatocyte ChREBPα on the activation of HSCs. Specifically, the conditioned medium was collected from hepatocyte Huh7 cells transduced with Ad‐Chrebpα versus Ad‐LacZ control after washing off the viruses. Adenoviral overexpression of ChREBPα in Huh7 was confirmed by immunoblotting (Figure S3, Supporting Information). Then, the medium was used to culture primary mouse hepatic stellate cells (pmHSCs) for 48 h (**Figure** **4**A) prior to the assessment of fibrosis markers including α‐SMA and Vimentin.^[^ ^4a^ ^]^

With the conditioned medium from Ad‐Chrebpα‐transduced hepatocytes, pHSCs showed reduced positive staining of α‐SMA and Vimentin (Figure 4B). In contrast, the positive staining of both markers was elevated when those cells were cultured in the conditioned medium from Ad‐shChrebpα‐transduced hepatocytes (Figure 4C). Of note, we observed the similar effects of conditioned medium on LX2 cells, a human hepatic stellate cell line (Figure S4, Supporting Information).

Based on the in vitro evidence from the medium transfer experiment, we further tested whether hepatocyte *Chrebpα* deficiency directly impacts HSC activation vivo (Figure 4D). To this end, we induced the early‐onset activation of HSCs by intraperitoneally injecting WT versus *Chrebpα‐LKO* mice with two doses of CCl4 before isolating primary HSCs. Immuno‐staining of primary HSCs for α‐SMA revealed three distinct groups among those cells: quiescent cells stained negative for α‐SMA; partially activated HSCs stained positive for α‐SMA without protrusions; fully activated HSCs with positive α‐SMA signal and protrusions. In the three WT mice injected with CCl4, ≈25% quiescent HSCs were identified along with 50–60% partially activated HSCs and 10–20% fully activated HSCs. This is in contrast with *Chrebpα‐LKO* mice showing less than 20% quiescent HSCs, 10–25% partially activated HSCs, and 60% fully activated HSCs, which were significantly higher than that in WT mice. These results indicate that hepatocyte *Chrebpα* deficiency creates a microenvironment favorable for HSCs activation in vivo (Figure 4E). Meanwhile, the mRNA levels of HSCs activation markers including *Col1a1, α‐SMA*, and *Vimentin* were also significantly elevated in pHSCs isolated from *Chrebpα‐LKO* mice (Figure 4F). In summary, we provide both in vitro and in vivo evidence supporting an anti‐fibrogenic role of hepatocyte ChREBPα against the activation of HSCs in liver.

### 2.5 Hepatocyte ChREBPα Communicates with HSCs via Secreted Factors THBS1 and CTGF

**Figure 5:**
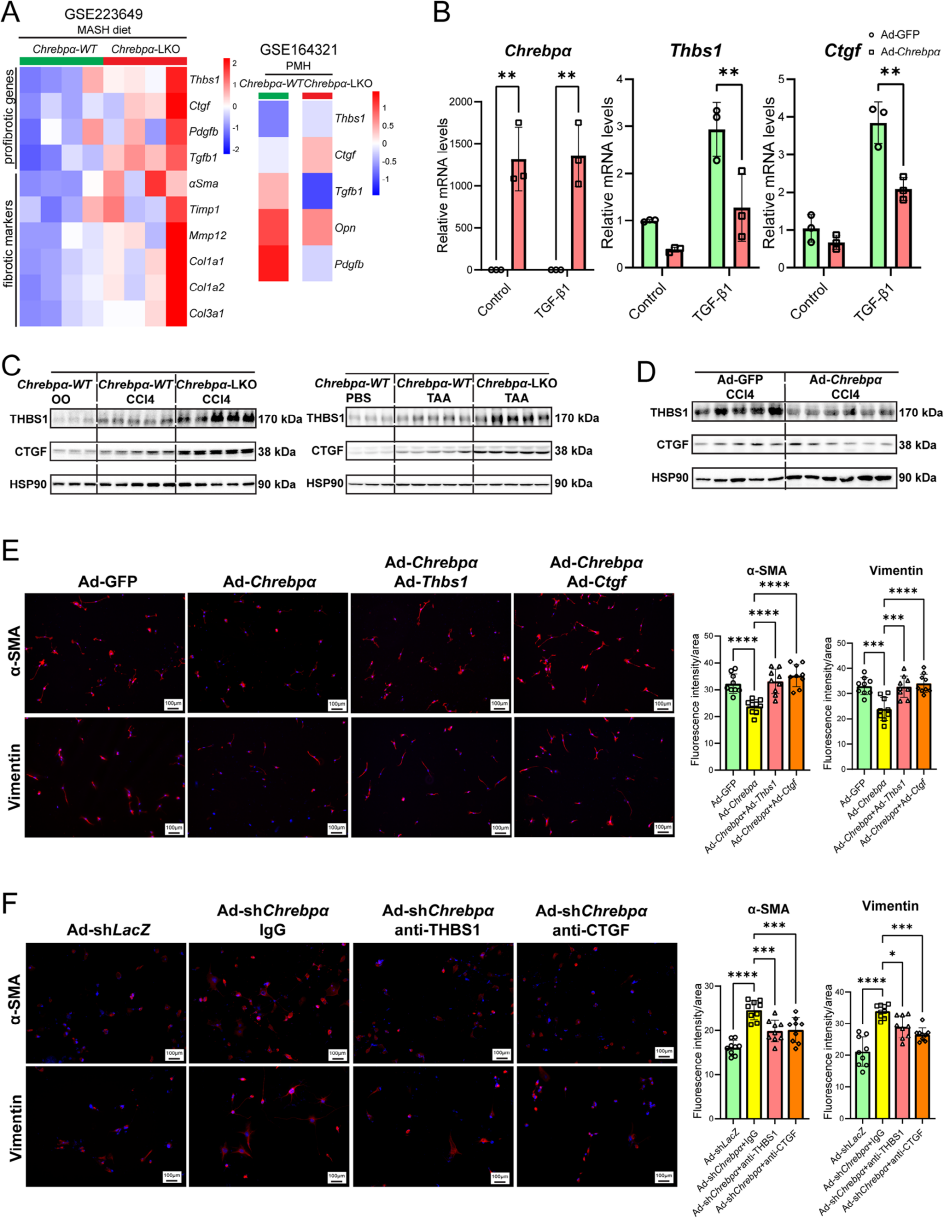
Overexpression of THBS1 and CTGF blocks hepatic ChREBPα‐mediated suppression of HSC. (A) Heatmap of significantly changed profibrotic genes and fibrotic markers in MASH diet‐induced *Chrebpα‐WT* and *Chrebpα‐LKO* mice (Left panel). Heatmap of microarray analysis in WT versus Chrebp‐/‐ primary mouse hepatocytes (Right panel). (B) The mRNA levels of *Chrebpα, Thbs1*, and *Ctgf* in WT PMHs transduced with Ad‐GFP versus Ad‐Chrebpα prior to TGF‐β1 (2 ng mL^−1^) treatment for 16 h. (C,D) Liver THBS1 and CTGF protein levels in previous three mouse models, including group#1:*Chrebpα‐WT* /Olive Oil group (*n* = 3), *Chrebpα‐WT* /CCl4 group (*n* = 5), *Chrebpα‐LKO /*CCl4 group (*n* = 5); group #2: Ad‐GFP/CCl4 group (*n* = 5), Ad‐Chrebpα /CCl4 group (*n* = 6); and group #3: Chrebpα‐WT/PBS group (*n* = 3), *Chrebpα‐WT* /TAA group (*n* = 5), *Chrebpα‐LKO* /TAA group (*n* = 5). (E) IF staining of α‐SMA and VIMENTIN in pmHSCs treated with conditioned medium derived from Huh7‐Ad‐GFP, Ad‐Chrebpα, Ad‐Chrebpα+Ad‐Thbs1, or Ad‐Chrebpα+Ad‐Ctgf (*n* = 9 fields/group). (F) IF staining and statistical results (*n* = 9 fields/group) of α‐SMA and VIMENTIN in pHSC treated with conditioned medium derived from Ad‐shLacZ, Ad‐shChrebpα with/without antibody‐neutralization by IgG, anti‐THBS1 or anti‐CTGF antibodies. All results are shown as Mean ± SD. All the invitro experiments were repeated at least once with similar results observed. RT‐qPCR results were analyzed by the two‐tailed Student's *t*‐test and immunofluorescence results were analyzed by one‐way ANOVA and followed with post‐hoc Tukey test. **p < 0.01, ***p < 0.001, and ****p < 0.0001.

Since both hepatocytes and HSCs were not in direct contact with one another in the medium transfer experiments, we speculated that hepatocyte ChREBPα is likely to communicate and modulate the activation of HSCs via secreting factor(s), most likely hepatocyte‐derived profibrogenic factors.^[^ ^24^ ^]^ Although reported studies suggest hepatocytes can crosstalk with HSCs via a panel of secreting factors, including cytokines, miRNA, and lipid molecules,^[^ ^5^, ^25^ ^]^ we focused on the profibrogenic factors based on our RNA‐Seq data from the liver samples from WT versus *Chrebpα‐LKO* mice after NASH diet feeding (GSE223649).^[^ ^11b^ ^]^ In the absence of hepatocyte *Chrebpα*, several profibrogenic factors are elevated, including *Thbs1*,^[^ ^26^ ^]^ *Ctgf*, ^[^ ^27^ ^]^ *Pdgfβ*,^[^ ^28^ ^]^ and *Tgf‐β1*.^[^ ^27a^ ^]^ Moreover, we found that both *Ctgf and Thbs1* but not *Pdgfβ and Tgf‐β1* were also upregulated in *Chrebp^−/−^* PMHs in our microarray analysis (GSE164321) (**Figure** **5**A).^[^ ^29^ ^]^ Although multiple factors are likely to contribute to the crosstalk, we focused on THBS1 (Thrombospondin‐1) and CTGF (Connective Tissue Growth Factor) because of their well‐established pro‐fibrotic roles in organ fibrosis. In PMHs, TGF‐β treatment potently induced the expression of *Ctgf* and *Thbs1*, whereas overexpression of *Chrebpa* effectively abrogated such an induction (Figure 5B). Of note, this is the first report showing the anti‐TGF‐b action of ChREBPa in hepatocytes.

Next, we examined the impact of *Chrebpα* manipulation in hepatocytes on the protein expression of THBS1 and CTGF in mouse models of liver fibrosis. In both CCl4 and TAA models, THBS1 and CTGF were elevated in the liver of WT mice and further enhanced in *Chrebpα‐LKO* mice (Figure 5C). In contrast, hepatocyte overexpression of ChREBPα reduced both THBS1 and CTGF in CCl4‐injected WT mice (Figure 5D). Taken together, these findings support 1) Hepatocyte ChREBPa antagonizes TGFb‐induced *Thbs1* and *Ctgf* mRNA in a cell‐autonomous manner; 2) Liver CTGF and THBS1 expression is inversely correlated with hepatocyte ChREBPα level. Thus, we hypothesized that THBS1 and CTGF could mediate the crosstalk between hepatocyte ChREBPα and HSCs.

To test this hypothesis, we first generated recombinant adenovirus expressing *Thbs1* and *Ctgf* and confirmed the overexpression of both by immunoblotting (Figure S5A,B, Supporting Information). With these adenoviruses, we generated four types of conditioned medium from transduced Huh7 cells: Ad‐GFP, Ad‐Chrebpα, Ad‐Chrebpα plus Ad‐Thbs1, and Ad‐Chrebpα plus Ad‐Ctgf. After being cultured in this conditioned medium, pHSCs were stained for α‐SMA and Vimentin by immunofluorescence. Consistent with previous findings, pHSCs cultured in Ad‐Chrebpα‐conditioned medium exhibited markedly reduced α‐SMA and Vimentin when compared with pHSCs cultured in Ad‐GFP‐conditioned medium. However, the intensity of α‐SMA and Vimentin signals was restored in pHSCs cultured in either Ad‐Chrebpα plus Ad‐Thbs1 or Ad‐Chrebpα plus Ad‐Ctgf versus Ad‐Chrebpα alone conditioned medium (Figure 5E).

Second, we employed antibody neutralization to eliminate THBS1 or CTGF in the conditioned medium collected from Adsh*Chrebp*α‐transduced Huh7 cells. The specificity of anti‐THBS1 and anti‐CTGF was validated by immunoprecipitation assay (Figure S6, Supporting Information). After incubation with either anti‐THBS1 or anti‐CTGF, the conditioned medium was used to culture pHSCs before immunofluorescence staining for α‐SMA and Vimentin. Consistently, pHSCs cultured in Ad‐shChrebpα conditioned medium showed elevated signals of α‐SMA and Vimentin, but the signal intensity of both proteins was significantly reduced in conditioned medium treated with anti‐THBS1 or anti‐CTGF (Figure 5F). Taken together, these results support that hepatocyte ChREBPα acts as a brake on the expression and secretion of THBS1 and CTGF that serve to mediate the crosstalk between hepatocytes and HSCs.

### 2.6 ChREBPα‐Mediated Antagonism of TGFβ‐Mediated Liver Fibrosis via Suppression of E2F1 In Vitro and In Vivo

Both *Thbs1* and *Ctgf* are well‐established transcriptional targets activated by TGF‐β signaling in hepatocytes.^[^ ^7^, ^8^, ^30^ ^]^ Interestingly, overexpression of ChREBPα abrogated TGFβ‐induction of *Thbs1* and *Ctgf* (Figure 5B). So far, how exactly ChREBPα could confer such an antagonism was unknown. We found that overexpression of *Chrebpα didn't* affect the total levels of SMAD2/3 or phosphorylated SMAD2/3, the classical marker of TGF‐β signaling (Figure S7, Supporting Information), suggesting that ChREBPα most likely antagonizes the TGF‐β actions via the SMAD‐independent pathways. Moreover, ChREBPα overexpression alone in mouse hepatocytes elevates SMAD7 and SMURF1, two negative regulators (Figure S8, Supporting Information). These results indicate that ChREBPα might promote the expression of known negative regulators of TGF‐β to block its induction of *Thbs1* and *Ctgf*.

**Figure 6:**
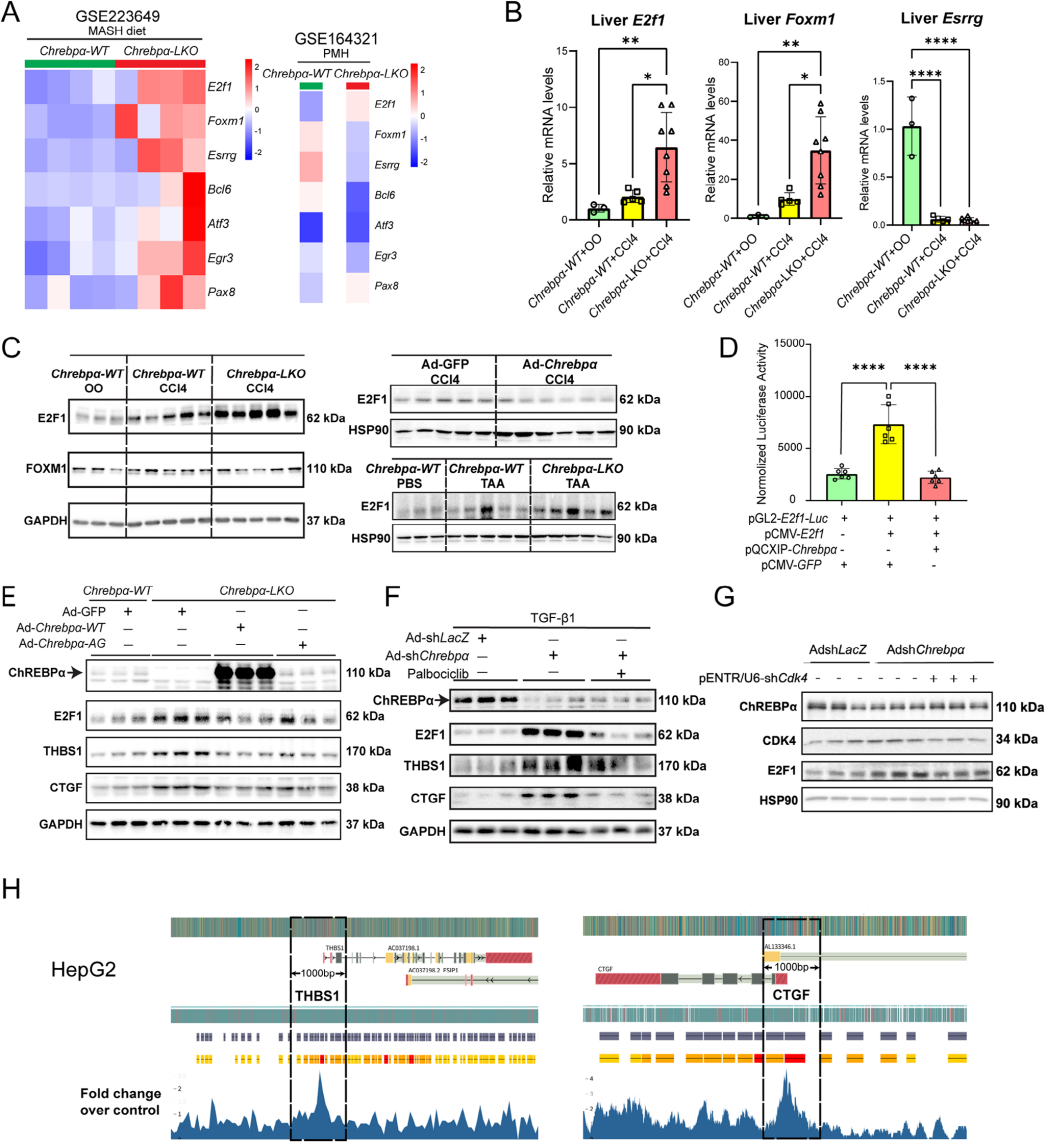
Hepatic ChREBPα suppresses THBS1 and CTGF via downregulating E2F1. (A) Heatmap of top changed fibrosis‐related transcriptional factors in MASH diet‐fed *Chrebpα‐WT* and *Chrebpα‐LKO* mice (Left panel). Heatmap of microarray analysis in WT versus *Chrebp^−/−^* primary mouse hepatocytes (Right panel). (B,C) The verification of top 3 changed transcriptional factors E2F1, FOXM1, ESSRG in previous three mouse models via RT‐qPCR and Immunoblotting. (D) The *E2f1‐luc* vector was co‐transfected with empty vector, pCMV‐E2f1 alone, or pCMV‐E2f1 & pQCIPX‐Chrebpα into 293A cells before harvested for luciferase assay. Luciferase activity was normalized by β‐Gal activity for each well. (E) Protein levels of ChREBPα, E2F1, THBS1, and CTGF in PMH from *Chrebpα‐WT* or *Chrebpα‐LKO* mice, then treated with Ad‐Chrebpα‐WT or Ad‐Chrebpα‐AG (*n* = 3/group). (F) Protein levels of ChREBPα, E2F1, THBS1 and CTGF in PMH treated with TGF‐β1 (2 ng mL^−1^), Ad‐shChrebpα, and CDK4 inhibitor, Palbociclib (*n* = 3/group). (G) Protein levels of ChREBPα, E2F1, and CDK4 in Hepa1c1c7 cells following transfection with shRNA against *Cdk4*. (H) Data mining of E2F1‐based ChIP‐seq for possible direct E2F1 binding to the 1 kb of TSS within the promoters of *THBS1* and CTGF promoter in HepG2 cell line (GSE169862). All results are shown as mean ± SD. All the invitro experiments were repeated at least once with similar results observed. Results were analyzed by one‐way ANOVA and followed with post‐hot Tukey test. *p < 0.05; **p < 0.01, ***p < 0.001, and ****p < 0.0001.

To gain further insights into the transcription factors downstream of ChREBPα that antagonize TGF‐β action, we therefore performed in‐depth analysis of our RNA‐Seq data (GSE223649) and microarray data (GSE164321)^[^ ^11^, ^29^ ^]^ and identified several fibrosis‐associated transcription factors, including *E2f1*,^[^ ^31^ ^]^ *Foxm1*,^[^ ^32^ ^]^ *Bcl6*,^[^ ^33^ ^]^ *Atf3*,^[^ ^34^ ^]^ *Esrrg*,^[^ ^35^ ^]^ *Pax8*,^[^ ^36^ ^]^ and *Egr3*,^[^ ^31a^ ^]^ that were upregulated in the liver of NASH diet‐fed *Chrebpα‐LKO* mice (**Figure** **6**A, left panel). Interestingly, only the *E2f1* mRNA was potently elevated in the *Chrebp^−/−^* PMHs (Figure 6A, right panel).

In the CCl4‐induced liver fibrosis model, hepatocyte deletion of *Chrebpα* led to a further increase of both *E2f1* and *Foxm1* mRNA levels in the liver of CCl4‐injected mice (Figure 6B). While only the E2F1 protein was significantly increased in the liver of CCl4‐injected *Chrebpα‐LKO* mice (Figure 6C left panel), its level was reduced in the liver of WT mice injected with both CCl4 and Ad‐Chrebpα (Figure 6C right top panel). We observed a similar elevation of the E2F1 protein in the liver of TAA‐injected *Chrebpα‐LKO* mice (Figure 6C right bottom panel), in support of ChREBPα as a negative regulator of E2F1 in liver fibrosis.

To further uncover the molecular mechanism by which ChREBPα represses the mRNA expression of *E2f1*, we first examined the effects of ChREBPα overexpression on the luciferase activity driven by a 275‐bp human *E2F1* promoter sequence.^[^ ^37^ ^]^ This reporter construct was significantly activated by E2F1 overexpression alone, consistent with the reported observation.^[^ ^37^ ^]^ However, the E2F1‐dependent induction of luciferase activity was potently repressed by ChREBPα overexpression, indicating that ChREBPα could directly suppress the transcription of *E2f1* by inhibiting its promoter activity (Figure 6D). Next, we test whether ChREBPα inhibits E2F1 expression in a hepatocyte‐specific manner, we isolated PMHs from WT or *Chrebpα‐LKO* mice for transduction with Ad‐GFP versus Ad‐Chrebpα to restore ChREBPα, which was confirmed by immunoblotting. The protein levels of E2F1, THBS1, and CTGF were elevated in Ad‐GFP‐transduced *Chrebpα‐LKO* PHMs but reduced in *Chrebpα‐LKO* PMHs with adenovirus‐mediated *Chrebpα* rescue (Figure 6E). To further test whether the DNA‐binding activity of ChREBPα is required for such an action, we generated the adenovirus expressing ChREBPα‐AG mutant, which was shown to be deficient in DNA binding and activating lipogenic gene expression^[^ ^11b^ ^]^ and observed a similar reduction of the levels of E2F1, TBHS1, and CTGF (Figure 6E). Taken together, we demonstrated that ChREBPα down‐regulates the E2F1 promoter activity and subsequently protein expression within hepatocytes.

The major regulatory mechanism for E2F1 expression and activity is through CDK4/6‐mediated phosphorylation of RB, an E2F1 repressor and binding partner.^[^ ^38^ ^]^ In animal models of obesity, elevated E2F1 pathway in the liver is likely to due to increased levels of CDK4/Cyclin D1.^[^ ^39^ ^]^ CDK4‐specific inhibitors have been developed for cancer treatment but not yet for metabolic diseases.^[^ ^40^ ^]^ To test whether this pathway is involved in ChREBPα‐induced suppression of E2F1 expression, we employed a well‐established CDK4 inhibitor Palbociclib in PMHs to block the CDK4 activity.^[^ ^41^ ^]^ In TGF‐β‐treated hepatocytes, we observed an induction of E2F1, THBS1, and CTGF. However, co‐treatment of Palbociclib effectively blocked the TGF‐β action on these proteins (Figure S9, Supporting Information). We then examined the effect of Palbociclib on E2F1 in the presence of *Chrebpα* depletion in PMHs. While we observed an increase of E2F1 by Ad‐shChrebp transduction in TNF‐treated PMHs, there was a clear drop of E2F1 in those cells after Palbociclib‐treatment (Figure 6F). In parallel, we used shRNA to specifically knockdown CDK4 in mouse hepatocytes and observed similar effects on E2F1 protein with Palbociclib (Figure 6G). Thus, we demonstrated that CDK4 is required for the induction of E2F1 by *Chrebpα* deficiency in hepatocytes via both pharmacological and genetic approaches.

To gain further insights into the molecular mechanisms by which E2F1 regulates the transcription of *Thbs1* and *Ctgf*, we performed analysis of the published E2F1 ChIP‐Seq database (GSE169862).^[^ ^42^ ^]^ In this study, the genomic‐wide E2F1 binding profile was performed in HepG2 cells with antibody against E2F1 versus control IgG followed by direct sequencing after anti‐E2F1 pulldown. Clear binding peaks were detected within 1 kb of the promoter regions from TSS for both *Thbs1* and *Ctgf*, supporting that E2F1 regulates the transcription of both genes by directly binding to their promoters (Figure 6H).

### 2.7 Acute Depletion of Liver E2F1 Reverses CCL4 and HFLMCD Induced Liver Fibrosis

**Figure 7:**
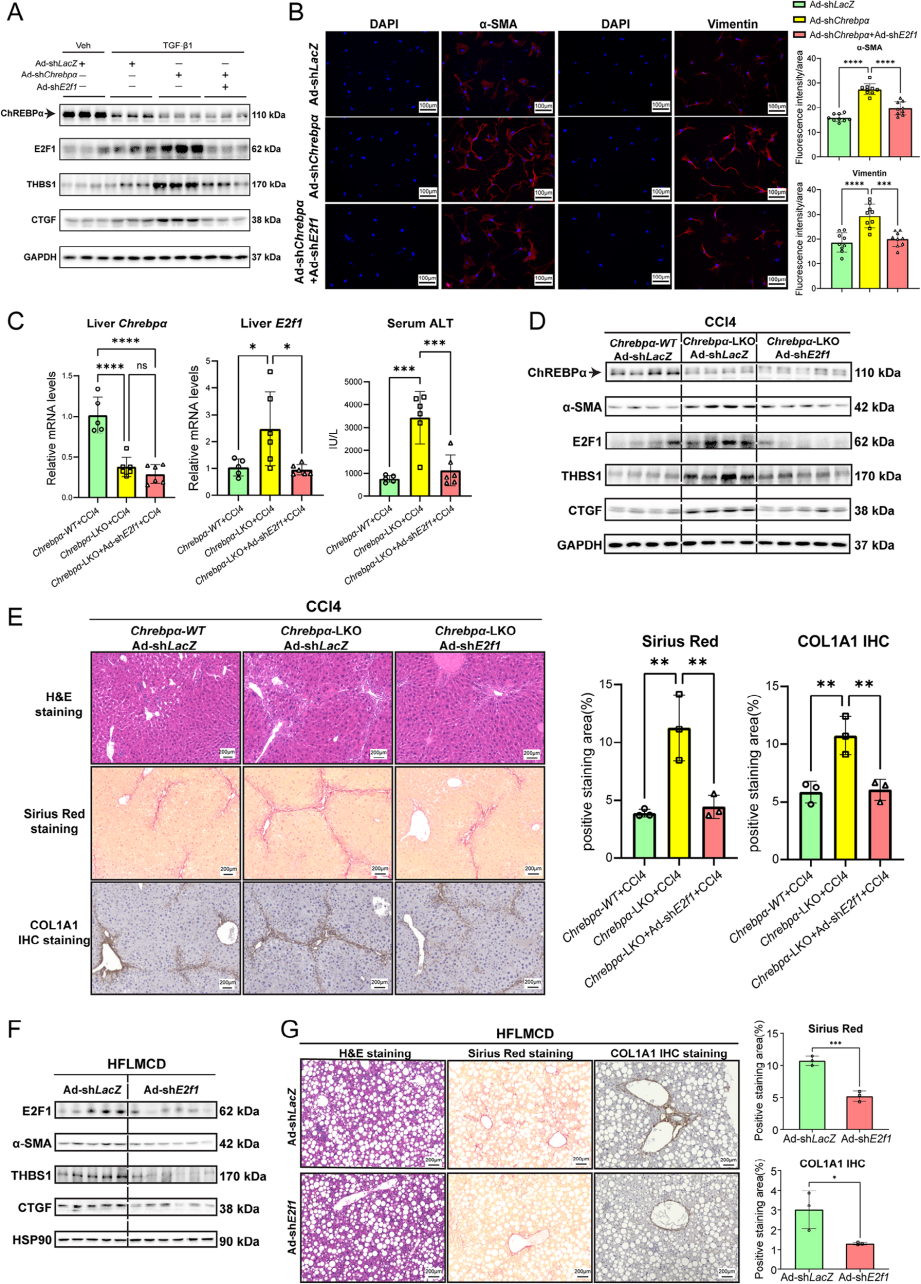
Depletion of hepatic *E2f1* attenuates *Chrebpα* deficiency‐enhanced liver fibrosis in response to CCl4 injection. (A) Protein levels of ChREBPα, E2F1, THBS1 and CTGF in PMHs treated with TGF‐β1, Ad‐shChrebpα, or Ad‐shE2f1 (*n* = 3/group). (B) IF staining and quantified results of α‐SMA and Vimentin in pHSC incubated in conditioned medium derived from Huh7 transduced with Ad‐shLacZ, Ad‐shChrebpα, or Ad‐shChrebpα+Ad‐shE2f1 (*n* = 9 fields/group). Effects of acute depletion of hepatic *E2f1* on CCl4‐induced liver fibrosis. (C) The mRNA levels of hepatic *Chrebpα* and *E2f1*, and serum ALT in CCl4‐treated *Chrebpα‐WT*/Ad‐shLacZ group (*n* = 5), *Chrebpα‐LKO*/Ad‐sh*LacZ* group (*n* = 6), and *Chrebpα‐LKO*/Ad‐sh*E2f1* group (*n* = 6); (D) Protein levels of liver ChREBPα, α‐SMA, E2F1, THBS1, and CTGF; (E) H&E staining, Sirius Red staining, and COL1A1 IHC staining of liver samples from these three groups and statistical results (*n* = 3/group). Effects of acute depletion of hepatic E2f1 on HFLMCD‐induced liver fibrosis. (F) Protein levels of liver E2F1, α‐SMA, THBS1, and CTGF in Ad‐shLacZ (*n* = 5) and Ad‐shE2f1 group (*n* = 6) fed with HFLMCD for 5 weeks. (G) H&E staining, Sirius Red staining, COL1A1 IHC staining of liver samples and statistical results (*n* = 3/group) from these two groups. All results are shown as mean ± SD. Differences between groups were analyzed with one‐way ANOVA or two‐tailed Student's *t‐*test. *p < 0.05; **p < 0.01, ***p < 0.001, and ****p < 0.0001.

To examine whether E2F1 is required for induction of THBS1 and CTGF in the absence of ChREBPα, we generated the adenoviral shRNA targeting *E2f1* and used it to deplete the endogenous *E2f1* in PMHs transduced with Ad‐shChrebpα versus Ad‐shLacZ. Consistent with previous findings, depletion of *Chrebpα* increased E2F1, THBS1, and CTGF levels. However, the simultaneous depletion of both *E2f1* and *Chrebpα* reduced THBS1 and CTGF levels (**Figure** **7**A), supporting that E2F1 is responsible for the induction of THBS1 and CTGF upon the loss of *Chrebpα*.

So far, we have demonstrated that hepatocyte ChREBPα antagonizes the TGFβ‐E2F1‐driven THBS1 and CTGF expression, leading us to speculate that depletion of hepatic *E2f1* might reverse HSCs activation and liver fibrosis in *Chrebpα‐LKO* mice. To test this notion, we collected conditioned medium from Huh7 transduced with Ad‐shLacZ alone, Ad‐shChrebp alone, or Ad‐shChrebp plus Ad‐shE2f1 to culture pHSCs for 48 h before immunofluorescence staining. The signal intensity of α‐SMA and Vimentin was all elevated in pHSCs cultured in Ad‐shChrebp versus Ad‐shLacZ conditioned medium. However, the conditioned medium from Huh7 cells with the simultaneous depletion of both *E2f1* and *Chrebpα* reduced the fluorescence signal of α‐SMA and Vimentin to the basal level (Figure 7B). These in vitro results support that hepatocyte ChREBPα suppresses the activation of HSCs via inhibiting E2F1.

To investigate this pathway in vivo, we challenged both *Chrebpα^f/f^* and *Chrebpα‐LKO* mice with CCl4 to induce liver fibrosis. After four rounds of injections of CCl4, mice were administered with Ad‐shLacZ or Ad‐shE2f1 via tail vein and followed with additional four CCl4 injections. At the end of experiment, the mRNA levels of liver *Chrebpα* and *E2f1* were measured by RT‐qPCR, validating the efficiency of hepatic *Chrebpα* deletion and *E2f1* knockdown. Serum ALT level was the highest in *Chrebpα‐LKO* mice injected with CCl4 plus Ad‐shLacZ, while it was markedly reduced in *Chrebpα‐LKO* mice injected with CCl4 plus Ad‐shE2f1 (Figure 7C). To analyze the degree of liver fibrosis in these groups of mice, we performed immunoblotting for α‐SMA, THBS1, and CTGF as well as Sirius Red staining for collagen and immunohistochemistry (IHC) for COL1A1. All these results showed that acute depletion of hepatic *E2f1* alleviates liver fibrosis in CCl4‐injected *Chrebpα‐LKO* mice (Figure 7D,E), supporting that hepatocyte ChREBPα‐specific anti‐fibrosis action is mediated via inhibiting E2F1 in vivo.

To further test whether inhibition of liver E2F1 also confers anti‐fibrosis action in diet‐induced MASH, we challenged mice with HFLMCD diet to induce liver inflammation and fibrosis prior to tail vein injection of Ad‐shLacZ versus Ad‐shE2f1. As shown in Figure 7F, we observed that mice injected with ad‐shE2f1 showed a reduction in total protein levels of E2F1, α‐SMA, THBS1 as well as CTGF. H&E staining showed a similar degree of liver steatosis after HFLMCD diet feeding, indicating that acute *E2f1* depletion does not impact liver steatosis. However, both Sirius Red staining and COL1A IHC showed reduced liver fibrosis upon hepatic *E2f1* depletion (Figure 7G). Interestingly, serum ALT level was similar between the two groups (data not shown), suggesting that the short‐term reduction in liver fibrosis is insufficient to reverse liver injury. Taken together, we demonstrate the anti‐fibrotic action of E2F1 inhibition in both chemical‐ and diet‐induced liver fibrosis.

## 3 Discussion

As the predominant cell type in the liver, hepatocytes have been shown to carry out an array of key metabolic functions. However, the role of hepatocytes in surveilling hepatic microenvironment and monitoring the activation status of HSCs remain largely unexplored. Here we uncovered a novel hepatocyte‐driven molecular mechanism underlying HSCs activation and liver fibrosis, which involves a cascade of transcription regulators involving ChREBPα and E2F1. Our study establishes hepatocyte ChREBPα as a potent anti‐fibrogenesis factor in chronic liver injury by antagonizing TGFb‐E2F1‐THBS1/CTGF signaling pathway, directly linking persistently overactive hepatocyte TGFβ signaling to liver fibrosis.

The major finding of this study is the identification of a physiological role of hepatocyte ChREBPα in suppressing HSCs activation and liver fibrosis. We showed that hepatic Chrebpa is reduced in several mouse liver fibrosis (CCl4, TAA, and BDL) models and human liver fibrosis due to HBV, NASH, alcohol liver disease, and primary biliary cholangitis.^[^ ^15^, ^16^, ^17^, ^18^ ^]^ We also identified *Chrebpα* as a previously unknown downstream target of TGFβ signaling in hepatocytes. Hepatocyte *Chrebpα* deficiency augmented hepatic fibrogenic responses caused by hepatotoxin (CCl4 or TAA) and cholestasis (BDL), providing direct evidence for its crucial role in the regulation of liver fibrosis following hepatocyte injury. More importantly, such an enhanced fibrogenic response is dissociated from hepatic lipid dysregulation, arguing that the newly discovered anti‐fibrosis function of hepatic ChREBPα is independent of its canonical lipogenic action. Interestingly, we previously reported a lipogenesis‐defective mutant of ChREBPα (ChREBPα‐AG) when overexpressed in liver, can protect mice from diet‐induced MASH and reduce liver fibrosis.^[^ ^11b^ ^]^ These lines of evidence strongly suggest that a non‐lipogenic ChREBPα could be a potential new therapeutic target to treat liver fibrosis without impacting lipid metabolism. Hormonal signals such as thyroid hormone and nutritional signals such as fructose and glucose are potent stimuli to induce ChREBPα expression.^[^ ^10^ ^]^ Unfortunately, they also promote ChREBPα‐driven lipogenic actions, leading to liver steatosis, which itself is a stress to hepatocytes. Thus, further research is warranted to figure out how to dissociate ChREBPα’s lipogenic action from its anti‐TGF‐β action. By selectively activating anti‐TGFβ function of ChREBPα without affecting lipogenesis could be an ideal avenue to treat liver fibrosis.

We also provided the evidence supporting hepatocyte ChREBPα monitors intrahepatic microenvironment and communicates with HSCs continuously. As suggested in Figure 4, most pHSCs isolated from CCl4‐injected *Chrebpα‐LKO* mice were fully activated, whereas the majority of WT pmHSCs remained quiescent, thus supporting the idea that hepatocyte *Chrebpα* deficiency promote a pro‐fibrogenic milieu in which pmHSCs are poised to respond to injury signals and become activated at an accelerated speed. At the molecular level, we found that hepatocyte ChREBPα suppresses TGF‐β‐induced profibrogenic factors, namely THBS1 and CTGF. Hepatocyte *Chrebpα* deficiency is sufficient to induce THBS1 and CTGF even without TGF‐β treatment. It is conceivable that the abundance of these profibrogenic factors creates a pro‐fibrogenic microenvironment in the liver of *Chrebpα‐LKO* mice. THBS1 has been shown to enhance TGFβ action in HSCs,^[^ ^26^, ^43^ ^]^ while CTGF has broad effects on HSCs proliferation, migration, and ECM production and remodeling.^[^ ^27^ ^]^ Inhibition CTGF pathway via siRNA, CTGF‐specific vaccines or nanobody have shown antifibrosis in various animal models with liver fibrosis.^[^ ^44^ ^]^ It remains unclear whether THBS1 could crosstalk with CTGF to synergistically activate HSCs. Interestingly, CTGF consists of several functional domains (IGFBP, VWC, TSP1, and CT domain), which allow various protein‐protein interactions.^[^ ^45^ ^]^ Of note, the TSP1 domain of CTGF was known as thrombospondin type 1 repeats, which was first identified in THBS1 protein. It is tantalizing to speculate that THBS1 could form a complex with CTGF via TSP1 repeats to promote latent TGF‐β action and evoke potent HSCs activation.

One of major findings from our study is that ChREBPα, a major lipid regulator, confers antagonism against the TGFβ‐driven profibrogenic program within hepatocytes. Our findings suggest that ChREBPα achieves this antagonism mainly via downregulating the transcription factor E2F1 instead of the classical SMAD signaling. The signaling crosstalk between ChREBPα and E2F1 is unexpected since the main function of E2F1 is related to cell cycle, apoptosis, and oxidative stress.^[^ ^38^ ^]^ Our data provide two possible mechanisms. On one hand, ChREBPα can inhibit the promoter activity of *E2f1*. On other hand, the CDK4‐specific inhibitor Palbociclib or genetic depletion of *Cdk4* blocks the effects of ChREBPα on E2F1 in vitro, in support of the role of CDK4‐dependent pathway. A recent study suggests that ChREBP transcriptionally inhibits Cyclin D1 in gastric cancer cells and the expression of ChREBP was reduced in gastric cancer, liver cancer, and colorectal cancer tissues.^[^ ^46^ ^]^ Thus, it is conceivable that the downregulation of ChREBP may also change the tumor microenvironment and impact the activity of tumor‐associated fibroblasts.

In summary, our results highlight that hepatocyte ChREBPα, in addition to its classical role in lipid metabolism, possesses a potent anti‐fibrosis action by directly modulating the intrahepatic microenvironment to suppress the activation of HSCs. We also show that hepatocyte ChREBPα is potently suppressed by TGF‐β signaling. Despite the well‐established role of TGF‐β in liver fibrosis and the potential of blocking TGFβ signaling to treat liver fibrosis, recent approaches to neutralize TGF‐β itself or inhibit its receptor all failed due to serious side effects. It is likely that ChREBPα could be a safer and more selective druggable target to treat liver fibrosis.

Our study contains several limitations. Although our antibody neutralization approach demonstrated that THBS1 and CTGF are both required to activate HSCs in the presence of hepatocyte *Chrebpα* deficiency, we have no evidence to determine how these two profibrogenic factors work in concert to stimulate HSCs activation. We only examined the outcome of short‐term *E2f1* depletion on liver fibrosis using adenovirus‐mediated shRNA, the long‐term hepatocyte‐specific *E2f1* deficiency on MASH and other types of liver fibrosis is still missing. Lastly, we showed that ChREBPα suppresses the E2F1 expression in a CDK4‐depdendent manner in cultured hepatocytes. However, in vivo genetic evidence to support the role of CDK4 in the suppression of E2F1 by ChREBPα is still lacking.

## 4 Experimental Section

### Animal Research

Chrebpα^f/f^ mice were generously provided by Dr. Lawrence Chan, Dr. Pradip Saha, and Dr. Alli Artar at Baylor College of Medicine. The study was approved by the Institutional Animal Care and Use Committee (IACUC) at the University of Michigan. C57BL/6 mice were housed under a 12‐h light/12‐h dark cycle with unrestricted access to food and water. Eight‐week‐old male littermates were injected intraperitoneally with CCl4 (Cat# 319961, Sigma–Aldrich, US) at a dose of 0.5 µL g^−1^ body weight twice a week for 4 weeks, or with TAA (Cat# 163678, Sigma–Aldrich, US) at a dose of 150 mg kg^−1^ body weight three times a week for 6 weeks, or fed with HFLMCD (45 kcal% Fat, 0.1% Methionine and Choline deficient diet, A06071309, Research Diets, US) for 5 weeks to induce liver fibrosis. Olive oil (Cat# O1514, Sigma–Aldrich, US) or PBS served as control solvents. Twelve‐week‐old male littermates underwent bile duct ligation (BDL) surgery to induce liver fibrosis and were euthanized for analysis after 7 days. Sham‐operated mice served as the control group. Liver‐specific knockout of Chrebpα was achieved via tail vein injection of AAV‐TBG‐GFP or AAV‐TBG‐CRE. For liver‐specific overexpression of Chrebpα and knockdown of E2f1, adenoviruses expressing GFP, Chrebpα‐WT, Chrebpα‐AG, shLacZ, or shE2f1 were delivered via tail vein injection.

### Immunofluorescent Staining

Immunofluorescence staining for αSMA and VIM was performed on methanol‐fixed cells. Samples were blocked with 10% normal goat serum for 30 min, then incubated with primary antibodies overnight at 4 °C. After washing, fluorophore‐conjugated secondary antibodies were applied for 1 h. Nuclei were stained, and slides were mounted with ProLong™ Gold Antifade reagent containing DAPI (Invitrogen, US). Average fluorescence intensity was normalized to total positive area size, and nine random fields per group were analyzed using Image‐Pro Plus 6.0 (NIH, Bethesda, US).

### Microarray and RNA‐Seq Analysis

All microarray, RNA‐seq data and ChIP‐seq data (GSE84044, GSE126848, GSE142530, GSE164321, GSE223649 and GSE169862) were obtained from the GEO database (https://www.ncbi.nlm.nih.gov/geo/).

### Cell Culture

Huh7, Hepa1c1c7, and LX2 cell lines were obtained from ATCC and maintained according to the manufacturer's protocols. Adenoviruses were added to the culture media after seeding. Cells were cultured in serum‐free media overnight prior to treatment. TGF‐β1 (2 ng mL^−1^) was used to induce liver fibrosis in vitro. SIS3 (3 µM, HY‐13013, MCE, US) was used to inhibit SMAD3, Palbociclib (1 µm, HY‐50767, MCE, US) to inhibit CDK4, MK‐2206 (65 nm, HY‐10358, MCE, US) to inhibit AKT, PD18416 (100 nm, HY‐10174, MCE, US) to inhibit ERK, and DMSO served as the solvent control.

### Primary Mouse Hepatocyte and Stellate Cell Isolation

Primary mouse hepatocytes (PMH) were isolated as described previously.^[^ ^47^ ^]^ Briefly, the liver was perfused with EBSS (Invitrogen, US) containing 0.5 mm EGTA, followed by perfusion with type I collagenase (Cat# LS004196, Worthington, US) via the inferior vena cava. Hepatocytes were dispersed, passed through a 100‐µm cell strainer, and spun before resuspension in DMEM. A Percoll gradient was used to remove dead cells. Primary hepatic stellate cells (pHSC) were isolated as described previously^[^ ^48^ ^]^ using protease (Cat# P5147, Sigma‐Aldrich, US) and collagenase D (Cat# 11088858001, Roche, Germany) via the inferior vena cava, followed by Nycodenz density gradient centrifugation.

### Generation of Plasmids and Adenovirus

AAV‐TBG‐CRE and AAV‐TBG‐GFP were purchased from the UPENN Vector Core. Adenoviruses including Ad‐shLacZ, Ad‐shChrebpα, Ad‐GFP, Ad‐Chrebpα‐WT, and Ad‐Chrebpα‐AG were previously described.^[^ ^11a,c^ ^]^ Construction of pGL2‐E2f1 luciferase reporter plasmid was the same as Neuman Group(5). The adenoviral vectors for E2f1, Thbs1, and Ctgf were constructed by subcloning the full‐length coding sequences into the pDONR/Zeo vector (Invitrogen, US). pAdEasy‐E2f1/Thbs1/Ctgf was generated through LR recombination with the pAdCMV‐GW vector (Invitrogen, US). The pEntry/U6‐shRNA plasmids were created by ligating dsDNA oligonucleotides with the pEntry/U6 vector (Invitrogen, US). The targeting sequences for the mouse and human E2F1 coding regions are 5′‐GGATCTGGAGACTGACCATCA‐3′ and 5′‐GCATCAGAGACCTCTTCGACT‐3′, respectively. The targeting sequences for the mouse CDK4 coding regions are 5′‐CTGCCGGTTGAGACCATTAAG‐3′. The AdBlock‐iT‐shE2f1 plasmid was generated via Gateway LR recombination between pEntry/U6‐shE2f1 and the pAdBlock‐iT vector (Invitrogen, US). All adenoviruses were produced in 293AD packaging cells.

### Statistics

Statistical analyses were performed using GraphPad Prism version 9.1 (GraphPad Software). Results are presented as mean ± SD. Two‐tailed Student's *t*‐test was used to compare two groups. Comparisons of three or more groups were made using one‐way or two‐way ANOVA followed with post‐hoc Tukey test. A *p*‐value < 0.05 was considered statistically significant.

Additional materials and methods are included in the Supporting Information.

## Conflict of Interest

The authors declare no conflict of interest.

## Author Contributions

J.Z. with help of Y.Z. carried all the animal experiments, performed tissue analysis, analyzed the data, and generated figures. J.Z. also drafted parts of manuscript. Q.Z. performed BDL surgery. J. Z., G. P., G.Z., and Y.Z. generated all the expression vectors and recombinant adenoviruses for in vitro and in vivo experiments with the guidance from X.T., J. Z., H.W., and J.S. G. performed primary hepatocyte isolation, primary hepatic stellate cells isolation, immunofluorescence staining, and biochemical analysis. L.Y. and X.T. supervised the project and wrote the manuscript. Inputs and suggestions from all authors were incorporated.

## Supporting information



Supporting Information

## Data Availability

The data that support the findings of this study are available from the corresponding author upon reasonable request.
