# Supplementary material for: The Non‐Canonical ChREBPα Activity Suppresses the Activation of Hepatic Stellate Cells and Liver Fibrosis by Antagonizing TGF‐β‐E2F1 Axis
Source: Adv Sci (Weinh). 2025 Jun 23;12(29):e15032. doi: 10.1002/advs.202415032 (PMC12362833; doi:10.1002/advs.202415032)
Supplement: Supplementary file 1 — Supporting Information [file ADVS-12-e15032-s001.docx]

**Supplementary information**

**Additional Materials and Methods**

**Biochemical Evaluation**
Serum alanine transaminase (ALT) levels were measured using a commercial kit (Cat# 23-666-089, Pointe Scientific, US) following the manufacturer's instructions. To assess hepatic lipids, liver tissues were weighed and homogenized in 1% acetic acid (7 μL acetic acid/mg liver tissue). After centrifugation, 200 μL of the supernatant was mixed with 800 μL of chloroform/methanol (2:1, v/v). The mixture was centrifuged at 10,000xg for 10 minutes at room temperature, and 450 μL of the organic phase was collected from the bottom layer. Samples were left in a fume hood overnight to evaporate. Total lipids were dissolved in 200 μL of ethanol, incubated at 55°C for 20 minutes, and 3 μL of the solution was used to measure triglycerides (Cat# 23-666-410) and cholesterol (Cat# 23-666-200, Pointe Scientific, US).

**Liver Histology Staining**
After fixation in 10% formalin, mouse liver samples were sent to the ULAM Pathology Core at the University of Michigan for paraffin embedding and hematoxylin and eosin (H&E) staining. For Sirius Red staining, deparaffinized and hydrated sections were stained with 0.1% picro-sirius red solution for 1 hour, washed with two changes of 0.5% acetic acid, dehydrated in ethanol, cleared in xylene, and mounted in a resinous medium. For immunohistochemistry (IHC), deparaffinized and hydrated sections underwent antigen retrieval in citrate buffer (pH 6.0) using a microwave for 20 minutes. Endogenous peroxidase activity was blocked with 3% hydrogen peroxide for 10 minutes. Sections were blocked in 10% normal goat serum for 30 minutes and incubated with the primary antibody at 4°C overnight. Secondary antibody incubation followed, and detection was carried out using DAB (Vector, US). Sirius Red, and IHC staining were quantified by randomly choosing three fields from captured images per liver specimen and the data were analyzed using Image-Pro Plus 6.0 (NIH, Bethesda, US).

**RNA Extraction and RT-qPCR**
Total RNA was extracted from fresh mouse liver tissue or hepatocytes using Trizol reagent (Invitrogen, US). Complementary DNA (cDNA) was synthesized using the Verso cDNA kit (ThermoFisher Scientific, US) and subjected to quantitative PCR (qPCR) using 2X Universal SYBR Green Fast qPCR Mix (Abclonal, US) on a QuantStudio 5 Real-Time PCR System (Applied Biosystems, Foster City, CA). Relative gene expression was analyzed using the 2^−ΔΔCt method.

**Western Blot Analysis**
Whole-cell lysates from hepatocytes or frozen liver samples were prepared using a RIPA buffer. Protein concentration was measured by the BCA protein assay. Equal amounts of protein were separated on 6%, 9%, or 12% SDS-PAGE gels, transferred to a nitrocellulose membrane, and blocked with 5% horse serum in TBST for 30 minutes at room temperature. The membrane was incubated with the primary antibody overnight at 4°C, followed by secondary antibody incubation for 1 hour. Chemiluminescent detection was performed.

**Plasmid Transfection and Luciferase Assay**

293T cells were plated in 12-well plates and allowed to adhere overnight. When cells density is about 80%, transfection was carried out using Opti-MEM (Gibco, 2021431) and polyethylenimine (PEI). Cells were co-transfected with the pGL2-E2f1 luciferase reporter plasmid, pCMV-E2f1 plasmid, and pQCXIP-Chrebpα or pCMV-GFP, along with the β-gal plasmid as an internal control. After 48 hours, luciferase activity was assessed using luciferin (Gold Biotechnology, 103404-75-7) on a BioTek Synergy 2 microplate reader and subsequently normalized to β-gal activity.

Antibodies used in the study

| Name | Company | Catalog | Host | Application and Dilution |
| --- | --- | --- | --- | --- |
| ChREBPα | Cell signaling | 58069S | Rabbit | WB (1:1000) |
| αSMA | ABclonal | A17910 | Rabbit | WB (1:1000); IF (1:200) |
| VIMENTIN | Cell signaling | 5741S | Rabbit | WB (1:1000); IF (1:200) |
| COL1A1 | Cell signaling | 72026T | Rabbit | IHC (1:200) |
| THBS1 | ABclonal | A2125 | Rabbit | WB (1:1000); IP (1:100) |
| CTGF | ABclonal | A11067 | Rabbit | WB (1:1000); IP (1:100) |
| E2F1 | Santa Cruz | sc-251 | Mouse | WB (1:800) |
| FOXM1 | Santa Cruz | sc-376621 | Mouse | WB (1:1000) |
| GAPDH | Santa Cruz | sc-32233 | Mouse | WB (1:1000) |
| HSP90 | Santa Cruz | sc-13119 | Mouse | WB (1:1000) |
| β-actin | ABclonal | AC026 | Rabbit | WB (1:1000) |
| p-AKT | Santa Cruz | sc-7985-R | Rabbit | WB (1:1000) |
| AKT | Cell signaling | 4691 | Rabbit | WB (1:1000) |
| p-ERK | Santa Cruz | sc-7383 | Mouse | WB (1:1000) |
| ERK | Santa Cruz | sc-94 | Rabbit | WB (1:1000) |
| p-SMAD3 | ABclonal | AP0548 | Rabbit | WB (1:1000) |
| SMAD3 | ABclonal | A11388 | Rabbit | WB (1:1000) |
| CDK4 | Santa Cruz | sc-56277 | Mouse | WB (1:1000) |

**Sequences of RT-qPCR primers**

| Gene | Forward | Reverse |
| --- | --- | --- |
| m-18S | TTGACGGAAGGGCACCACCAG | GCACCACCACCCACGGAATCG |
| m-β-actin | TTAATTTCTGAATGGCCCAG | GACCAAAGCCTTCATACATC |
| m-Gapdh | ATGTTCCAGTATGACTCCACTC | GAAGACACCAGTAGACTCCACGACA |
| m-αSMA | CCTTCGTGACTACTGCCGAG | TATAGGTGGTTTCGTGGATGCC |
| m-Tgfβ | GCTGAACCAAGGAGACGG | ATGTCATGGATGGTGCCC |
| m-Chrebpα | GAAGCCACAGTGAAATCTCG | TTGTTCAGCCGGATCTTGTC |
| m-E2f1 | CTCGACTCCTCGCAGATCG | GATCCAGCCTCCGTTTCACC |
| m-Foxm1 | CTGATTCTCAAAAGACGGAGGC | TTGATAATCTTGATTCCGGCTGG |
| m-Esrrg | AAGATCGACACATTGATTCCAGC | CATGGTTGAACTGTAACTCCCAC |
| m-Col1a1 | GAGGCCTCCCCAGAACATCAC | GAGGCCTCCCCAGAACATCAC |
| m-Col1a2 | AGTCGATGGCTGCTCCAAAA | AGCACCACCAATGTCCAGAG |
| m-Thbs1 | TGACAATTTTCAGGGGGTGCT | AGAAGGACGTTGGTAGCTGAG |
| m-Ctgf | CGCCAACCGCAAGATTG | ACACGGACCCACCGAAGAC |
| m-Smad7 | GGCCGGATCTCAGGCATTC | TTGGGTATCTGGAGTAAGGAGG |
| m-Smurf1 | AGCATCAAGATCCGTCTGACA | CCAGAGCCGTCCACAACAAT |
| m-Smurf2 | AAACAGTTGCTTGGGAAGTCA | TGCTCAACACAGAAGGTATGGT |
| m-Bambi | GATCGCCACTCCAGCTACTTC | GCAGGCACTAAGCTCAGACTT |
| m-Dact2 | TCACGGCTAAGGAGACAGGAT | GATAGACGGTCGCTGCAAAC |
| m-Fkbp1a | GATTCCTCTCGGGACAGAAACA | GACCCACACTCATCTGGGCTA |
| m-Ppp1cc | GAGAACGAGATCCGAGGACTC | CGTATTCAAACAGACGGAGCAA |
| m-Fasn | TTGGCCCAGAACTCCTGTAG | CTCGCTTGTCGTCTGCCT |
| m-Acc1 | GAAGCCACAGTGAAATCTCG | GATGGTTTGGCCTTTCACAT |
| m-L-pk | CTGGAACACCTCTGCCTTCTG | CACAATTTCCACCTCCGACTC |
| m-Atpcl | CCTCAAGGACTTCGTCAAACA | GCCCATACTCCTTCCTAGCAC |
| m-G6pc | TCTGGGTGGCAGTGGTCGGA | CAGAGGGACTTCCTGGTCCGGT |
| m-Rgs16 | GATCCGATCAGCCACCAAAC | TGGTAGTGGCAGCTTGTAGG |
| m-Txnip | CCTTGATCTGCCCCTAGTGA | GGAGGAGCTTCTGGGGTATC |
| h-Gapdh | GGAGCGAGATCCCTCCAAAAT | GGCTGTTGTCATACTTCTCATGG |
| h-Chrebpα | CAGCTGCGGGATGAGATTGA | AAACGCTGGTGTGTGATGGGTA |
| h-E2f1 | CATCCCAGGAGGTCACTTCTG | GACAACAGCGGTTCTTGCTC |
| h-Thbs1 | TCCCCGTGGTCATCTTGTTC | GCCTCCATTGATGGGGCAG |
| h-Ctgf | CACCCGGGTTACCAATGACA | GGATGCACTTTTTGCCCTTCTTA |

**Supplementary Figures & Figure legends**

**Supplementary Figure 1** The quantification of Sirius Red staining and COL1A1 IHC staining results after CCl4 / TAA injection or BDL surgery in Chrebpα-WT and Chrebpa-LKO mice. The data were plotted as mean ±SEM. *<0.05 & **<0.01 by Student's t test.

**Supplementary Figure 2** Lipid metabolism in CCL4 or TAA induced liver fibrosis mouse model in Chrebpα-WT and Chrebpa-LKO group. (A) Liver triglyceride and liver cholesterol levels, (B) The mRNA levels of Chrebpα targets involved in lipid metabolism in Chrebpαf/f and Chrebpα-LKO mice injected with CCl4 for 4 weeks (n=3, 5, 8). (C) The mRNA levels of Chrebpα targets involved in lipid metabolism in Chrebpαf/f and Chrebpα-LKO mice injected with TAA for 6 weeks (n=4, 6, 6). The data were plotted as mean ± SD. *<0.05 & **<0.01 & *** < 0.001 & ****< 0.0001 by one-way ANOVA **.**

**
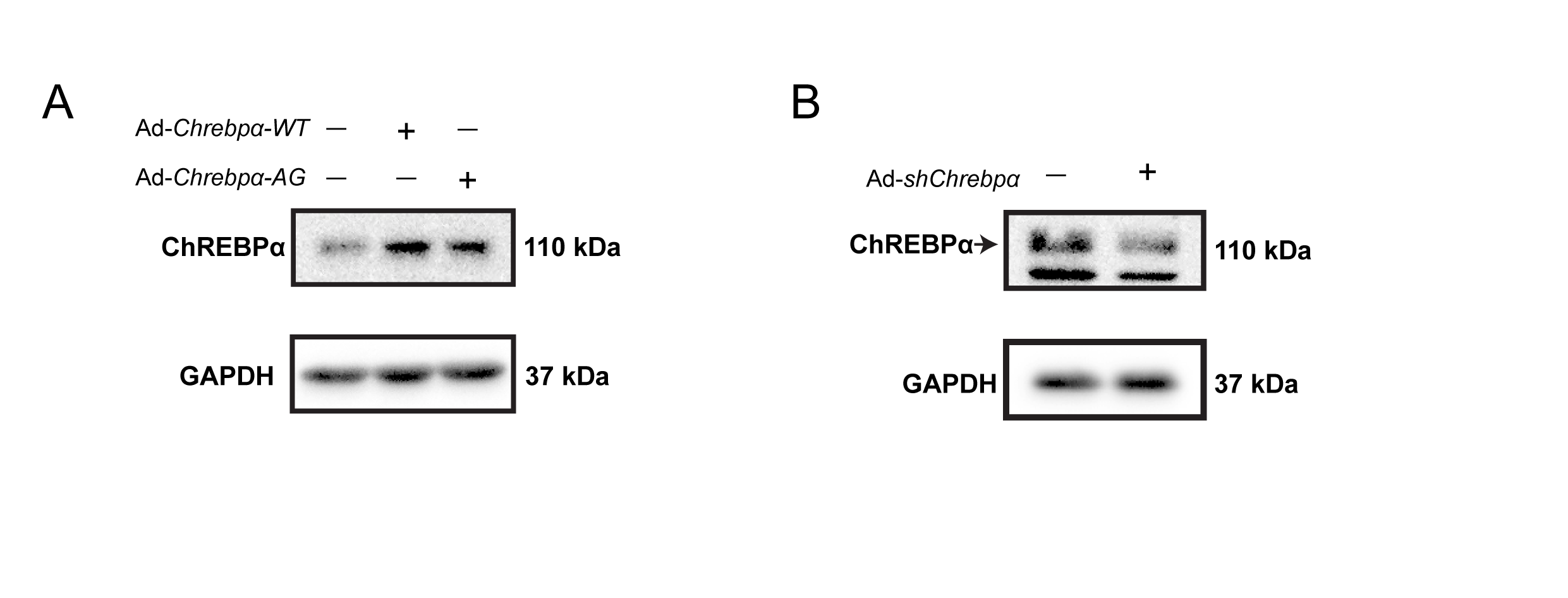
**

**Supplementary Figure 3** Validation of Adenovirus for either overexpressing or knocking down of ChREBPα in Huh7 cells**. (A)** Immunoblotting of ChREBPα in Huh7 transduced with Ad-Chrebpα-WT or Ad-Chrebpα-AG; **(B)** Immunoblotting of ChREBPα in Huh7 transduced with either Ad-sh*lacZ* or Ad-sh*Chrebpα*.

**
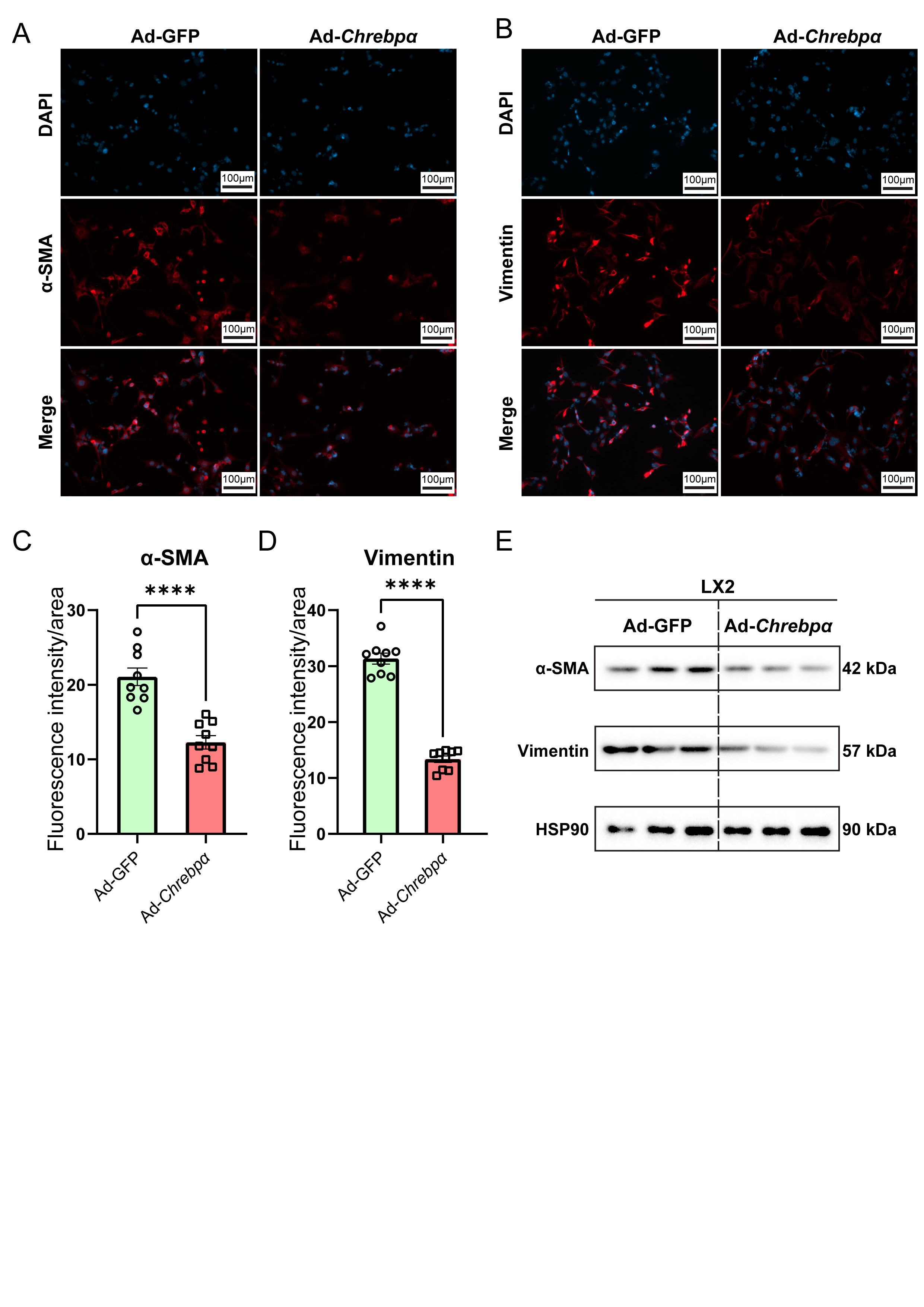
**

**Supplementary Figure 4** Effects of hepatocyte ChREBPα overexpression on activation of LX2 following medium transfer experiment. LX2 cells were incubated with conditioned medium derived from Huh7 transduced with either Ad-GFP or Ad-Chrebpα prior to immunofluorescence staining or immunoblotting analysis. **(A-B)** immunofluorescence staining of a-SMA and Vimentin; **(C-D)** Quantification of fluorescence intensity; **(E)** Protein levels of a-SMA and Vimentin by immunoblotting.

**
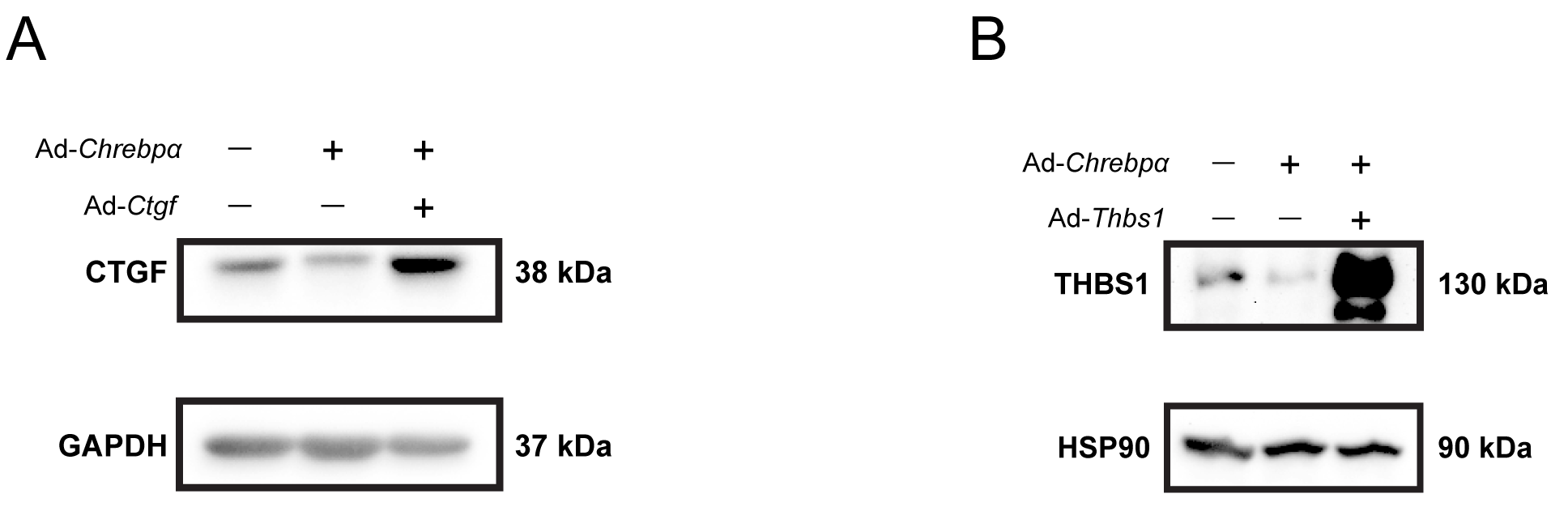
**

**Supplementary Figure 5** Validation of adenovirus for overexpressing CTGF and THBS1. The immunoblotting analysis of CTGF and THBS1 in Huh7 transduced with either Ad-Ctgf **(A)** or Ad-Thbs1**(B).**

**
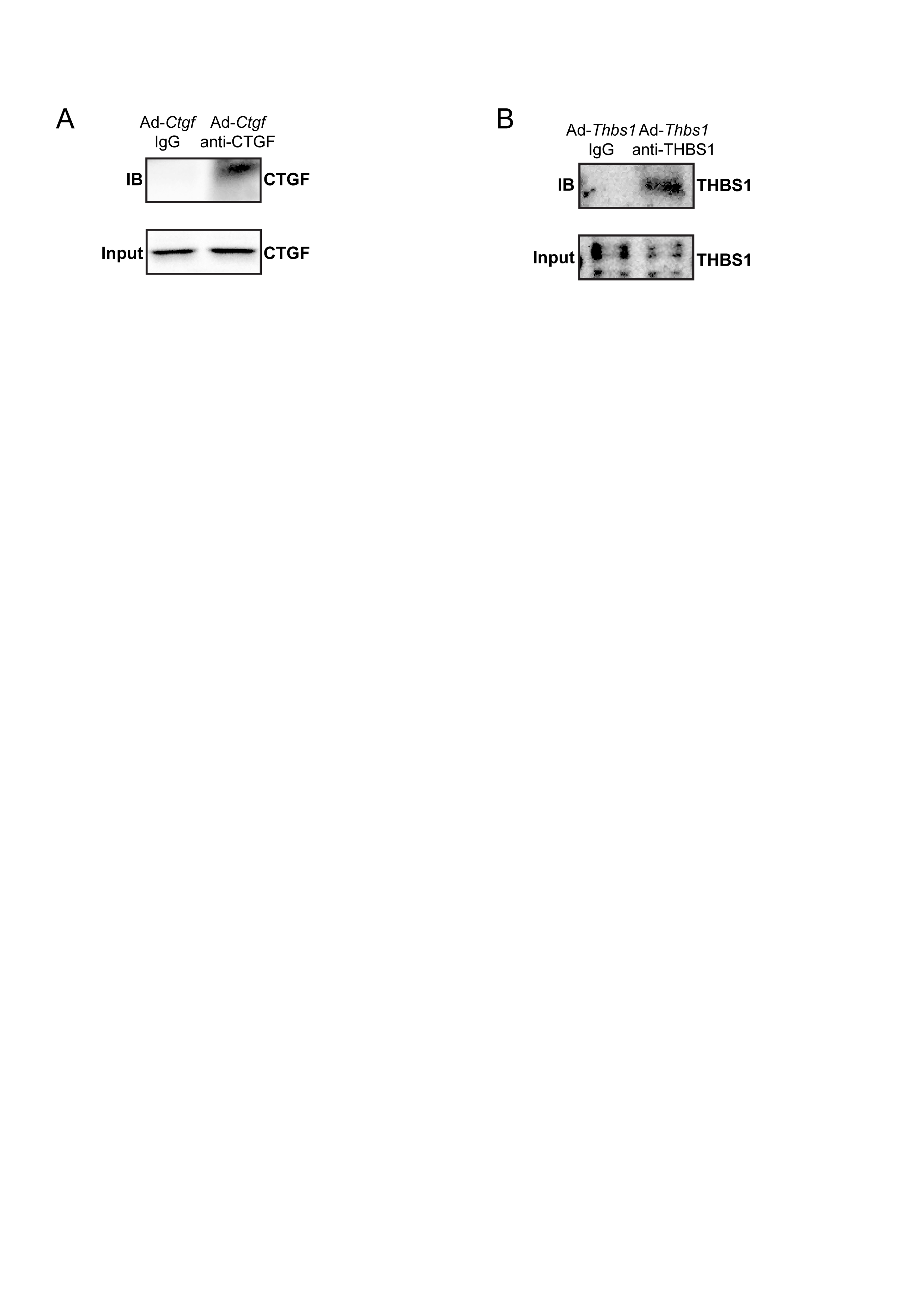
**

**Supplementary Figure 6** Confirming the specificity of anti-CTGF and anti-THBS1. Huh7 cells were firstly transduced with either Adcon or Ad-Ctgf prior to immunoprecipitation assay with Anti-CTGF. The presence of CTGF protein after pull-down assay was determined by immunoblotting with anti-CTGF (ABclonal, 1:100) (A). The same approach was used to determine specificity of anti-THBS1(ABclonal, 1:100) (B).

**
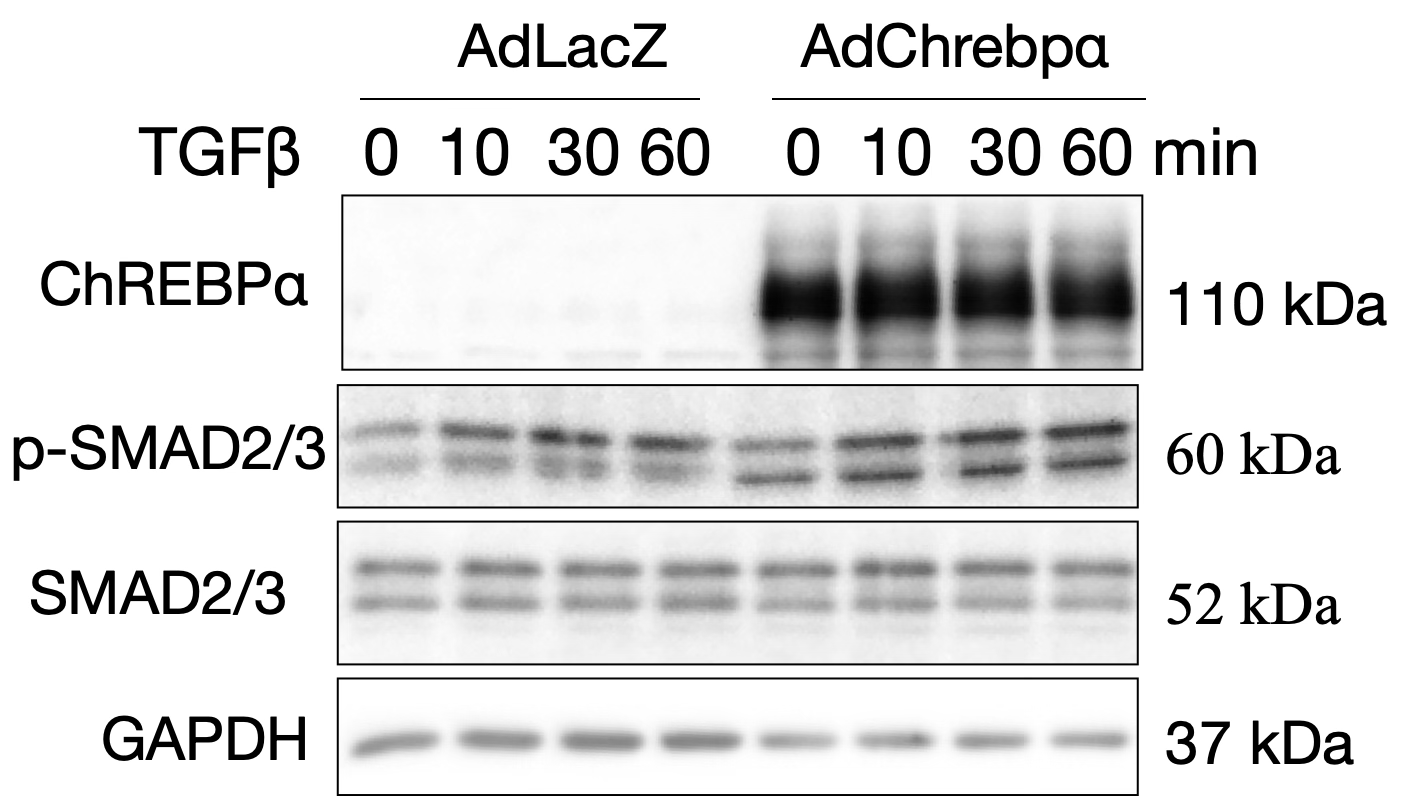
**

**Supplementary Figure 7** Effects of hepatocyte ChREBPα overexpression on TGF𝛽 induced SMAD2/3 phosphorylation. Primary hepatocytes were transduced with Ad-LacZ or Ad-Chrebpα. After 16 hours serum-free MEM medium incubation, cells were treated with TGF𝛽(1ng/ml) for 0, 10, 30, 60 minutes then subjected to western blotting.

**Supplementary Figure 8** Effects of hepatocyte ChREBP*α* overexpression on TGF𝛽 negative regulators**.** Primary hepatocytes were isolated from WT mouse and transduced with Ad-LacZ or Ad-Chrebp𝛼-WT or Ad-Chrebp𝛼-AG. The mRNA levels of genes related to TGF𝛽 negative regulators were examined by RT-qPCR. The data were plotted as mean ± SEM (n=4). *<0.05 & **<0.01 & *** < 0.001 & ****< 0.0001 by one-way ANOVA .

**
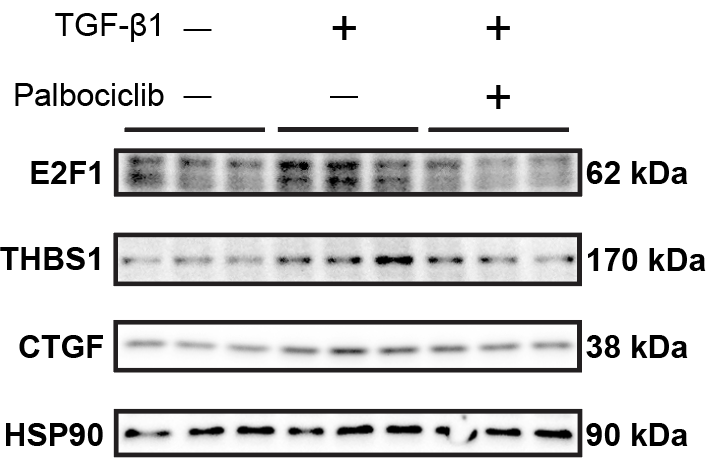
**

**Supplementary Figure 9** Palbociclib effectively blocked the TGF-β induced E2F1, CTGF, THBS1 expression. Protein levels of E2F1, THBS1 and CTGF in PMH treated with TGF-β1 (2 ng/mL) or the CDK4 inhibitor Palbociclib were detected by western blotting(n=3/group).
